# Aging concomitantly reduces skeletal muscle proteome plasticity and hypertrophic responses to resistance training in male adults

**DOI:** 10.14814/phy2.71141

**Published:** 2026-10-11

**Authors:** Dustyn T. Lewis, J. Max Michel, Mason C. McIntosh, Dakota R. Tiede, Daniel L. Plotkin, Madison L. Mattingly, Nicholas J. Kontos, George Kontos, Breanna J. Mueller, Samuel C. Norton, Joshua S. Godwin, Brad J. Schoenfeld, Melissa D. Boersma, Andrew D. Frugé, C. Brooks Mobley, Andreas N. Kavazis, Michael D. Roberts

**Affiliations:** ^1^ School of Kinesiology Auburn University Auburn Alabama USA; ^2^ Department of Exercise Science and Recreation, Applied Muscle Development Laboratory CUNY Lehman College Bronx New York USA; ^3^ Department of Chemistry and Biochemistry Auburn University Auburn Alabama USA; ^4^ College of Nursing Auburn University Auburn Alabama USA; ^5^ Edward Via College of Osteopathic Medicine – Auburn Campus Auburn Alabama USA

**Keywords:** aging, hypertrophy, proteomics, resistance training, skeletal muscle

## Abstract

Resistance training (RT)‐induced hypertrophy is attenuated with aging, albeit the proteomic basis of this attenuation remains unclear. We thus determined whether reduced skeletal muscle proteome plasticity accompanies age‐related blunted hypertrophic responses. Seventeen younger (21.9 ± 2.5 years) and 15 older (57.5 ± 6.9 years) untrained males completed 10–12 weeks of RT, and the vastus lateralis (VL) soluble proteome was profiled pre‐ and post‐training using data‐independent acquisition mass spectrometry (2106 proteins). Despite similar training volume (*p* = 0.771), younger adults gained more lean body mass (+2.73 vs. +1.18 kg, *p* = 0.009) and VL thickness (+0.49 vs. +0.20 cm, *p* = 0.002) than older adults. Proteome plasticity paralleled this divergence, as RT altered 135 proteins (FDR < 0.05) in younger adults and none in older adults. This attenuation was one of magnitude rather than direction, as 81.5% of the 135 younger‐adult proteins shifted concordantly in older adults (*ρ* = 0.711), and 75 gene sets were enriched with RT in older adults, led by translational machinery (NES up to +2.85). At baseline, 278 proteins differed between age groups (FDR <0.05), and age dominated the co‐expression network (Turquoise module AUC = 0.96). In conclusion, the blunted hypertrophic response of older males coincides with a loss of RT‐induced proteome plasticity, reflecting a compressed rather than qualitatively altered RT adaptation.

## Graphical abstract

We examined the vastus lateralis (VL) muscle proteomic signature in younger and older males before and after a 10–12‐week resistance training period. Despite similar training volumes between age groups, VL hypertrophy (determined via ultrasound) was dampened in older individuals, and this coincided with an attenuated proteome plasticity response. We also reveal that baseline proteomic state may constrain adaptive capacity, with molecular features distinguishing aging muscle potentially limiting its hypertrophic response. Nonetheless, older participants showed an observable reversal of proteomic signatures, with ribosomal proteins and proteins involved in translation most affected.

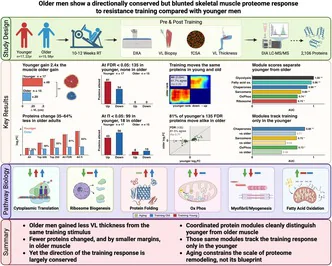

## 1 INTRODUCTION

Maintaining or increasing skeletal muscle mass is imperative among middle‐aged and older adults for reducing the risk of life‐threatening injuries from falls, cardiometabolic disease, and enhancing quality of life (Goodpaster et al., 2006; Srikanthan & Karlamangla, 2014; Wolfe, 2006). Skeletal muscle mass typically peaks in the third to fourth decade and declines progressively thereafter, with losses accelerating after age 60 (Cruz‐Jentoft, Bahat et al., 2019). This age‐related loss (i.e., sarcopenia) affects up to 45% of adults over 70 years, representing a major public health concern with attributed healthcare costs of $40.4 billion annually (Bijlsma et al., 2013; Gungor & Balci, 2026). Although resistance training (RT) is a viable strategy to maintain or increase muscle mass and strength, the hypertrophic responses are attenuated in older adults. This is evident in diminished changes in lean body mass relative to younger adults after equivalent training (Peterson et al., 2010; Peterson et al., 2011) compounded by selective type II fiber atrophy that accounts for the majority of age‐related muscle loss (Nilwik et al., 2013). This dissociation between preserved strength, achieved partly through enhanced neural adaptations (i.e., voluntary activation and motor unit recruitment) (Siddique et al., 2022; Walker & Hakkinen, 2014), and attenuated hypertrophy (i.e., anabolic resistance) represents a fundamental barrier to exercise‐based interventions for sarcopenia prevention (Gungor & Balci, 2026).

At the molecular level, several mechanisms contribute to the attenuated hypertrophic response in older adults, many of which reflect a broader loss of proteostasis, a primary hallmark of biological aging (Lopez‐Otin et al., 2023). Aging may blunt activation of the mTORC1 pathway in response to mechanical loading and amino acid availability, reducing muscle protein synthetic rates (Paulussen et al., 2021), though the magnitude and consistency of these deficits remain debated (Fuqua et al., 2023; Markofski et al., 2015). Age‐related deficits in autophagy‐lysosomal and ubiquitin–proteasome systems impair protein quality control and promote accumulation of damaged proteins (Jiao & Demontis, 2017). Age‐related deficits in mitochondrial oxidative capacity may also constrain bioenergetic resources needed for hypertrophic remodeling (Coen et al., 2018). Satellite cell dysfunction, characterized by a type II fiber–specific decline in satellite cell content and impaired activation in response to loading (Snijders et al., 2009; Verdijk et al., 2014), may compromise regenerative capacity. Similarly, reported deficits in cumulative protein synthesis coupled with attenuated ribosomal biogenesis may contribute to impaired RT responses in older individuals (Brook et al., 2016).

We put forth a recent hypothesis to suggest that aging is associated with reduced “proteome plasticity” to RT (Roberts et al., 2024); this being a term we use herein to denote the capacity of the muscle proteome to dynamically respond to anabolic stimuli, operationalized as the number, magnitude, and functional coherence of training‐induced protein abundance changes. Several lines of evidence support this concept. For instance, baseline proteomic differences between younger and older muscle are substantial (Deane et al., 2023; Gueugneau et al., 2014; Murgia et al., 2017; Roberts et al., 2024; Staunton et al., 2012; Ubaida‐Mohien et al., 2019; Vann et al., 2020), including mitochondrial metabolism, molecular chaperones, and cytoskeletal elements. These baseline differences could factor into the diminished training adaptations and constrained plasticity of the aged proteome. Chronic transcriptomic responses to RT are similarly dampened in older adults, with seven‐fold fewer responsive transcripts in older type II fibers (Raue et al., 2012). Nevertheless, RT can partially reverse age‐related gene expression signatures (Melov et al., 2007). Pre‐training transcriptomic profiles predict response heterogeneity (Thalacker‐Mercer et al., 2013), and ribosomal production predicts hypertrophy in older adults (Stec et al., 2016), suggesting that aging constrains both transcriptional and translational plasticity. However, direct comparisons of training‐induced proteome remodeling between younger and older adults remain scarce.

Among the limited studies examining RT‐induced proteomic changes across age groups, limitations include designs that did not train both age groups for direct comparison (Roberts et al., 2024), an emphasis on muscle function (rather than size changes) and proteomic outcomes (O'Leary et al., 2025), or limited dynamic range detection with first generation proteomic interrogation techniques (Deane et al., 2023). Deep proteomics studies of exercise‐induced skeletal muscle adaptations have arisen recently with notable contributions from the MoTrPAC consortium (MoTr, Lead et al., 2024), and independent research laboratories including our own (Hulmi et al., 2025; Jacques et al., 2025; Murgia et al., 2017; O'Leary et al., 2025; Roberts et al., 2024; Robinson et al., 2017).

The present investigation was motivated by the sparse evidence indicating that RT‐induced proteome remodeling may differ between younger and older adults. Herein, the soluble proteome of vastus lateralis (VL) biopsies was profiled in 17 younger (21.9 ± 2.5 years) and 15 older (57.5 ± 6.9 years) untrained males before and after 10–12 weeks of supervised RT using data‐independent acquisition mass spectrometry (2106 quantified proteins). We hypothesized that skeletal muscle proteome plasticity in response to RT would be constrained in the older versus younger cohort. We further hypothesized that age‐related constraints would extend to the pathway level, manifesting as discordance in training adaptations between age groups. Drawing on evidence that RT can partially reverse aging gene expression signatures, we hypothesized that RT might similarly reverse age‐related proteome signatures, suggesting that at least some age‐related alterations represent modifiable molecular phenotypes rather than irreversible features of aging. Beyond differential expression, we sought to identify trajectory clusters of co‐regulated proteins and to determine whether baseline proteomic profiles predict training‐induced hypertrophic outcomes.

## 2 MATERIALS AND METHODS

### 2.1 Participants and ethical approval

This study is a secondary analysis of VL muscle biopsies obtained from three parent investigations conducted at Auburn University (McIntosh et al., 2026; Ruple et al., 2021; Sexton et al., 2021). All parent studies were approved by the Auburn University Institutional Review Board (Protocol numbers 19–249, 20–136, & 24–863) and conducted in accordance with the Declaration of Helsinki, and two studies (20–136 & 24–863) were not preregistered as clinical trials. All participants provided written informed consent prior to enrollment.

A total of 17 younger (21.9 ± 2.5 years, range: 19–26 years) and 15 older (57.5 ± 6.9 years, range: 42–69 years) untrained males were included. All participants had refrained from structured resistance training for ≥6 months prior to enrollment, had a body mass index between 18.5 and 35 kg/m^2^, had no contraindications to muscle biopsy or resistance exercise, and were not using medications known to affect muscle metabolism (e.g., corticosteroids, testosterone, growth hormone). Two individual timepoint samples from separate younger participants were flagged and removed by a four‐method consensus (per‐sample missingness, principal component analysis (PCA) Mahalanobis distance, median‐intensity MAD, and inter‐sample Pearson correlation), with the unpaired partner from each participant retained, yielding 62 analyzed samples from 17 younger and 15 older adults. Participant characteristics are presented in Table 1, and excluded samples are identified in Table S8. As a secondary analysis, sample sizes were determined by the parent investigations. Post hoc analysis (pwr.t.test; 80% power, *α* = 0.10) indicated that the paired within‐group contrasts could detect effects of *d* ≥ 0.65 (log_2_FC ≥0.37), while the unpaired Aging and Interaction contrasts required *d* ≥ 0.93 (log_2_FC ≥0.63) (Table S10).

**TABLE 1:** Participant characteristics before and after resistance training.

Variable	*n*	Young pre	Young post	Old pre	Old post	*p* age	*p* time	*p* age × time
DXA lean body mass (kg)	17/15	56.6 ± 5.7	59.3 ± 6.4	58.3 ± 6.5	59.5 ± 7.1	0.679	<0.001	0.010
DXA fat mass (kg)	17/15	19.4 ± 9.0	20.1 ± 9.8	30.2 ± 6.7	30.0 ± 6.3	0.001	0.517	0.163
Fat‐to‐lean mass ratio	17/15	0.34 ± 0.15	0.33 ± 0.14	0.52 ± 0.11	0.51 ± 0.10	<0.001	0.071	0.658
VL thickness (cm)	17/15	2.08 ± 0.60	2.57 ± 0.47	1.95 ± 0.41	2.15 ± 0.40	0.100	<0.001	0.002
Type I fCSA (μm^2^)	13/10	4510 ± 1110	5084 ± 1250	5378 ± 1476	4655 ± 1457	0.668	0.743	0.008
Type II fCSA (μm^2^)	13/10	5422 ± 1609	6560 ± 1659	4541 ± 1330	4160 ± 944	0.008	0.116	0.003
Deadlift 1RM (kg)	17/9	100.3 ± 22.0	134.9 ± 27.0	95.8 ± 31.7	108.3 ± 26.6	0.150	<0.001	<0.001

Abbreviations: DXA, dual‐energy x‐ray absorptiometry; fCSA, fiber cross‐sectional area; FM, fat mass; LBM, lean body mass (bone‐free); VL, vastus lateralis.

### 2.2 Training protocol

All participants completed supervised, progressive resistance training programs targeting major muscle groups. Training was conducted twice per week over 10–12 weeks under direct supervision by trained research personnel. Specific training durations and programming details have been described in the respective parent investigations. Briefly, sessions included lower‐body exercises (45° leg press, leg extensions, lying leg curls, and squat or hex‐bar deadlift variations) and upper‐body exercises (chest press variations and cable pull‐downs or rows). Participants performed 3–5 sets of 6–15 repetitions per exercise with progressive overload guided by ratings of perceived exertion or repetitions in reserve. Volume load (sets × repetitions × load) was tracked throughout the intervention and compared between age groups. Volume load, lean body mass, and VL thickness were available for all participants (*n* = 17 younger, *n* = 15 older).

### 2.3 Vastus lateralis muscle thickness

VL muscle thickness was assessed using B‐mode ultrasound (NextGen LOGIQe R8; GE Healthcare; Chicago, IL, USA) with a multifrequency linear‐array transducer (L4‐12T, 4–12 MHz, GE Healthcare). Briefly, participants lay supine on an athletic training table while a technician oriented the probe in the transverse plane on the lateral aspect of their thigh to obtain still images of the VL. The pre‐intervention biopsy scar was used as a guide for replicating pre‐ and post‐intervention measurements. Ultrasound settings were held constant across participants for each individual study. VL muscle thickness was quantified by measuring the linear distance from the deep aponeurosis to the superficial interface between the VL and the subcutaneous adipose tissue in ImageJ (National Institutes of Health; Bethesda, MD, USA).

### 2.4 Body composition assessment

The height and weight of all participants was assessed using a digital column scale (Seca 769; Hanover, MD, USA). Prior to body composition testing, participants submitted a urine sample (≥5 mL) which was assessed for urine specific gravity using a handheld refractometer (ATAGO; Bellevue, WA, USA). Across studies, urine specific gravity was ≤1.030 for all participants analyzed. Participants then laid supine for an assessment of body composition via dual‐energy X‐ray absorptiometry (DXA) (Lunar Prodigy; GE Corporation; Fairfield, CT, USA) from which bone‐free lean body mass (LBM) was derived. Whole‐body fat mass was derived from the same DXA scans and is reported alongside lean mass (Table 1; Figure S2). Relative adiposity is reported as the fat‐to‐lean mass ratio rather than percent body fat: body mass was recorded only at baseline in one parent trial, so a percent‐of‐weight measure at post‐training would rest on a derived denominator for 11 of the 32 participants, whereas both DXA compartments were measured at both timepoints.

### 2.5 Tissue collection and processing

VL muscle biopsies were obtained at baseline (PRE) and ≥72 h following the final training session (POST) using the 5‐gauge percutaneous needle biopsy technique with suction. The mid‐belly of the right VL was identified at the midpoint between the inguinal crease and proximal border of the patella, locally anesthetized with 1% lidocaine, and approximately 50–100 mg of tissue was collected. Immediately following collection, muscle samples were cleared of visible blood, adipose, and connective tissue, and snap‐frozen in liquid nitrogen. Samples were stored at −80°C until processing. For proteomic analysis, the soluble protein fraction was extracted using a 1X general tissue lysis buffer (Cell Signaling Technology; Danvers, MA, USA; catalog #: 9803) containing 20 mM Tris–HCl (pH 7.5), 150 mM NaCl, 1 mM Na2EDTA, 1 mM EGTA, 1% Triton, 2.5 mM sodium pyrophosphate, 1 mM beta‐glycerophosphate, 1 mM Na3VO4, 1 μg/mL leupeptin. Briefly, ~20 mg muscle was minced on a liquid nitrogen‐cooled mortar and placed in 1.7 mL tubes on ice containing 500 μL of buffer. Tissue was mechanically homogenized with tight‐fitting pestles, and samples were centrifuged at 500×*g* for 5 min. Following centrifugation, supernatants (termed “lysates” hereafter) were placed in new tubes and frozen at −80°C until peptide generation. Notably, this lysis procedure tends to solubilize membrane, cytosolic, nuclear, and some contractile and extracellular matrix proteins according to prior assessments from our laboratory (Godwin et al., 2023; Roberts et al., 2020). This is not a single‐compartment preparation, however: clearing at 500 × g retains most myofibrillar protein (~80% of muscle protein on a dry‐mass basis) in the pellet, so the analyzed fraction is depleted of myofibrillar and over‐represented for sarcoplasmic protein relative to whole muscle. We therefore describe it as a soluble proteome rather than a sarcoplasmic‐enriched fraction.

### 2.6 Proteomic analysis

Protein lysates (50 μg per sample) were prepared for LC–MS/MS analysis using EasyPep Mini MS Sample Prep Kit (Thermo Fisher; Waltham, MA, USA; catalog #: A4006). Briefly, samples were transferred into new 1.7 mL microcentrifuge tubes and final volumes were adjusted to 100 μL with general cell lysis buffer (Cell Signaling). Reduction (50 μL) and alkylation (50 μL) solutions were added to samples, gently mixed, and incubated at 95°C using a heat block for 10 min. After incubation, the sample was removed from the heat block and cooled to room temperature. A reconstituted enzyme Trypsin/Lys‐C Protease solution (25 μL) was added to the reduced and alkylated protein sample and incubated with shaking at 37°C for 2 h to digest proteins. Digestion stop solution (25 μL) was then added to samples and peptides were cleaned using a peptide clean‐up column according to the kit instructions.

An externally calibrated Thermo Orbitrap Exploris 240 (high‐resolution electrospray tandem mass spectrometer) was used in conjunction with Dionex UltiMate3000 RSLC Nano System (Thermo Fisher) for proteomics. Samples were aspirated into a 20 μL loop and loaded onto the trap column (Acclaim PepMap 100 nanoviper tubing 75 μm i.d. × 2 cm; Thermo Fisher). The flow rate was set to 300 nL/min for separation on the analytical column (Easy‐Spray pepmap RSLC C18 50 μM × 15 cm; Thermo Fisher). Mobile phase A was composed of 99.9% H2O (EMD Omni Solvent; Millipore, Austin, TX, USA), and 0.1% formic acid and mobile phase B was composed of 80% acetonitrile and 0.1% formic acid. A 135‐min linear gradient from 3% to 50% B was performed. The eluent was directly nanosprayed into the mass‐spectrometer. During chromatographic separation, the Orbitrap Exploris was operated in a data‐independent mode and under direct control of the Thermo Xcalibur 4.4.16.14 (Thermo Fisher). The MS data were acquired from (380 to 985 m/z) at 60,000 resolution. MS2 spectra were acquired in data‐independent acquisition mode with a 10 m/z isolation window, 1 m/z overlap, collision energy of 28, and a resolution of 15,000. All measurements were performed at room temperature, and three technical replicates were run for each sample. Raw.dia files acquired from mass spectrometry were processed using DIA‐NN (v 2.2.0) (Demichev et al., 2020). All raw files were analyzed jointly in a single batch to enable retention‐time alignment and match‐between‐runs (MBR) quantification. In silico digestion was performed with trypsin‐P specificity, allowing one missed cleavage. Carbamidomethylation of cysteine was specified as a fixed modification, while oxidation of methionine was included as a variable modification, with three variable modification permitted per peptide. N‐terminal methionine excision was enabled. Peptide identification was guided by the DPHLv2 spectral library (Xue et al., 2023). For protein reannotation, a reviewed *Homo sapiens* UniProt reference proteome (release 2020‐04) supplemented with indexed retention‐time (iRT) calibration peptides, reversed decoys, and common contaminants were used. Peptide and protein‐level false discovery rates were controlled at 1%. Data‐independent acquisition (DIA) was chosen for its superior quantitative reproducibility and depth in skeletal muscle proteomics (Frohlich et al., 2024).

Proteins annotated as expressed in skeletal muscle (Human Protein Atlas, release 25.0, downloaded 6 February 2026) were deduplicated by UniProt accession and retained if detected in ≥10 samples within at least one experimental group. Highly abundant blood proteins and common contaminants were removed. Sample outliers were identified by a four‐method consensus (per‐sample missingness, PCA Mahalanobis distance, median‐intensity MAD, and inter‐sample Pearson correlation); two individual timepoint samples were removed and the unpaired partners retained (excluded samples identified in Table S8). Abundances were normalized by cyclic loess via the proteoDA R package (Thurman et al., https://github.com/ByrumLab/proteoDA). After filtering, 2106 proteins were retained for downstream analysis. The quality‐control pipeline is summarized in Figure S1a (normalization) and Figure S1b (imputation), with the panel‐level source data in Table S1. Normalized (Table S8) and imputed (Table S9) protein matrices are provided as Supplementary Data.

### 2.7 Statistical analysis

Differentially expressed protein (DEP) abundance was assessed using the limma R package with empirical Bayes moderation (Ritchie et al., 2015; Smyth, 2004). The model accounted for the paired PRE–POST design using the duplicateCorrelation function to estimate within‐subject correlation. Four contrasts were defined: Training in Young (Young_Post − Young_Pre), Training in Old (Old_Post − Old_Pre), Aging at Baseline (Old_Pre − Young_Pre), and Age × Training Interaction ([Old_Post − Old_Pre] − [Young_Post − Young_Pre]). Significance was determined using the Π‐score, which is a composite metric integrating both statistical significance and fold‐change magnitude (Π = *p* ^|log2FC|^), with proteins considered significant at *Π* < 0.05. Significance was also assessed by Benjamini–Hochberg‐corrected false discovery rate (FDR <0.05) and nominal *p*‐value (*p* < 0.05) to provide complementary thresholds. Missing values were imputed with missForest (ntree = 100, maxiter = 10) (Stekhoven & Buhlmann, 2012), selected from a 16‐method benchmark that scored candidate methods against the non‐imputed reference on fold‐change preservation, pathway‐level concordance, and reconstruction error; missForest was chosen for its strong composite performance, established literature precedent, and straightforward implementation (Table S9). Differential abundance testing was performed on non‐imputed data, as limma fits each protein independently using only available observations. Imputed values were employed where a complete data matrix was required (PCA, WGCNA). Additionally, the fry gene‐set rotation test (limma::fry) was used to assess whether pre‐defined DEP sets showed concordant or reversed behavior in an independent contrast, using the same paired structure as the differential expression fit (subject blocking with the duplicateCorrelation estimate); fry is self‐contained, so it tests whether a set responds rather than whether it responds more than the rest of the proteome. Module‐trait associations from weighted gene co‐expression network analysis (WGCNA) were assessed via linear mixed models (eigengene ~ group + (1|subject)) with Kenward‐Roger degrees of freedom to account for the paired design (Kenward & Roger, 1997; Langfelder & Horvath, 2008), and effect sizes reported as equivalent correlations derived from the model t‐statistics. Parent‐study supplementation was not entered into the design given that no parent trial contributed both age groups, so every supplementation arm lies wholly within one age group and a supplement term would be confounded with age rather than adjust for it (see Section 4.7).

#### 2.7.1 Protein set enrichment analysis (PSEA)

Two primary approaches were used to convey pathway‐level information. First, ranked‐list enrichment (fgseaMultilevel) tested proteins ordered by the limma moderated t‐statistic against MSigDB Hallmark, KEGG Medicus, Reactome, GO:BP, and GO Slim gene sets (size 15–500; disease, cancer, and infection‐related terms excluded), with Benjamini–Hochberg correction applied per database (Subramanian et al., 2005). Second, concordance and reversal analyses compared contrast‐level NES values across an a priori Hallmark and GO Slim collection. Over‐representation analysis (fgsea::fora) was applied to proteins partitioned by scatter‐plot quadrant with Benjamini–Hochberg correction and Jaccard deduplication (cutoff = 0.5) to reduce redundancy among overlapping gene sets (Liberzon et al., 2015).

#### 2.7.2 Concordance analysis

To assess whether younger and older adults exhibit concordant proteomic responses to training, a Spearman rank correlation was computed between Training Effect logFC values across all quantified proteins. Proteins were further classified into four concordance categories based on the significance and direction of their training response in each age group. Threshold‐free concordance was assessed using Rank‐Rank Hypergeometric Overlap (RRHO2), which partitions proteins into quadrants of concordant and discordant regulation without reliance on significance cutoffs (Liberzon et al., 2015). Global proteomic composition was visualized by principal component analysis on the imputed matrix, and the contribution of age, training time point, and their interaction was quantified by permutational multivariate analysis of variance (PERMANOVA; adonis2, 999 permutations) on Euclidean distances (Anderson, 2001). Per‐contrast effect‐size distributions were compared using pairwise Wilcoxon rank‐sum tests on absolute log_2_ fold‐change (log_2_FC) values.

#### 2.7.3 Reversal analysis

Each protein's aging effect (Old_Pre − Young_Pre) was compared with its training response in older adults (Old_Post − Old_Pre) and classified as reversed, exacerbated, or negligible (|log_2_FC| < 0.2); DEPs were further split into four directional categories. Because these contrasts share Old_Pre with opposite signs, their correlation is negative before any biology enters (Oldham, 1962; Smyth & Altman, 2013). Three coupling‐aware statistics were therefore computed: a within‐participant Pre/Post label‐swap null (1000 draws); a split‐half estimate in disjoint halves of the older participants (500 draws); and correlation with the younger‐adult training response, which shares no samples. Protein‐label permutation is reported for comparison only.

#### 2.7.4 Software and data availability

All statistical analyses and visualizations were performed in R (version 4.6.0). Key packages included limma, proteoDA, missForest, fgsea v1.38.0, msigdbr v26.1.0, GO. db v3.23.1, org.Hs.eg.db v3.23.1, WGCNA, ggplot2, and patchwork. Gene sets were retrieved from MSigDB release 2026.1.Hs, which redistributes KEGG and Reactome as C2 subcollections, with Gene Ontology terms and GO Slim assignments from GO release 2026‐01‐23. Code and processed data are available at https://github.com/Dustyn‐T‐Lewis/YvO_2026. Source data for all main figures are provided in Tables [Link], [Link], [Link], [Link], [Link], [Link].

## 3 RESULTS

### 3.1 Participant characteristics and training outcomes

Volume load was similar between age groups (*p* = 0.771; Figure 1a). Both age groups increased DXA‐derived lean body mass following training (Young: +2.73 kg, Older: +1.18 kg; between‐group *p* = 0.009; Figure 1b) and VL thickness (Younger: +0.49 cm, Old: +0.20 cm; Figure 1c). Young adults gained significantly more VL thickness than older adults (Δ = 0.284 cm, *p* = 0.002), confirming a differential hypertrophic response at the vastus lateralis despite comparable training stimulus. Younger adults also showed greater gains in deadlift one‐repetition maximum (interaction *p* < 0.001; *n* = 17 young, 9 old) and Type II fiber cross‐sectional area (interaction *p* = 0.003; *n* = 13 young, 10 old). Group means and per‐participant values are in Table S2. Older adults carried more fat mass than younger adults at baseline (30.2 ± 6.7 vs. 19.4 ± 9.0 kg, *p* = 0.001) and a higher fat‐to‐lean mass ratio (0.52 ± 0.11 vs. 0.34 ± 0.15, *p* < 0.001), and neither measure changed appreciably with training in either group (fat mass age × time *p* = 0.163; fat‐to‐lean *p* = 0.658; Table 1, Figure S2).

**FIGURE 1:**
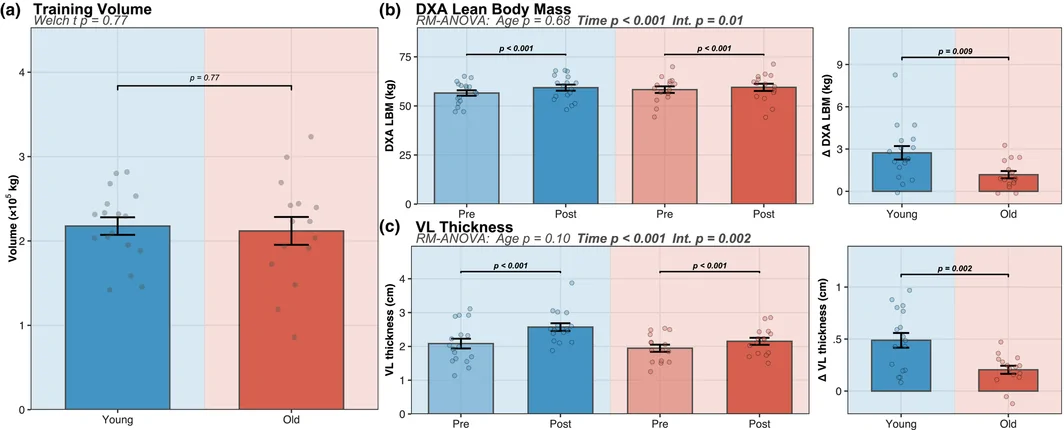
Phenotypic responses to resistance training in younger (*n* = 17) and older (*n* = 15) adults. (a) Volume load; groups were similar (*p* = 0.771). (b) DXA lean body mass pre‐ and post‐training (left) and between‐group change (right); both groups gained LBM (Young *p* < 0.001; Old *p* < 0.001); between‐group Δ was significant (*p* = 0.009). RM‐ANOVA: Age *p* = 0.679, Time *p* < 0.001, Interaction *p* = 0.010. (c) VL thickness pre‐ and post‐training (left) and between‐group change (right); younger adults gained more than older adults (Δ = 0.284 cm, *p* = 0.002). RM‐ANOVA: Age *p* = 0.100, Time *p* < 0.001, Interaction *p* = 0.002. Values are means ± SE. DXA, dual‐energy X‐ray absorptiometry; LBM, lean body mass; VL, vastus lateralis.

### 3.2 Proteomics summary

After quality‐control filtering, 2106 proteins were quantified across 62 VL biopsies from 17 younger and 15 older adults sampled before and after resistance training. Median inter‐individual coefficients of variation ranged from 23% to 26% across all four groups (Figure S3). Baseline CVs were comparable between age groups, though training induced modest but significant shifts in variability, with the largest divergence occurring post‐training. Principal component analysis revealed partial separation by age group, though not by training status (Figure 2a). PERMANOVA nonetheless detected significant main effects of age (*R* ^2^ = 0.089, *p* = 0.001) and training time point (*R* ^2^ = 0.029, *p* = 0.001), while the age × time interaction was non‐significant (*R* ^2^ = 0.013, *p* = 0.260). Effect‐size distributions differed substantially across contrasts (all pairwise *p* < 0.001, Wilcoxon rank‐sum on | log_2_FC|; Figure 2b). The Aging contrast exhibited the broadest spread (median |log_2_FC| = 0.18), followed by Training in Young (0.13) and Training in Old (0.09), revealing progressive compression of per‐protein effect sizes from the aging signature to the training response in older adults.

**FIGURE 2:**
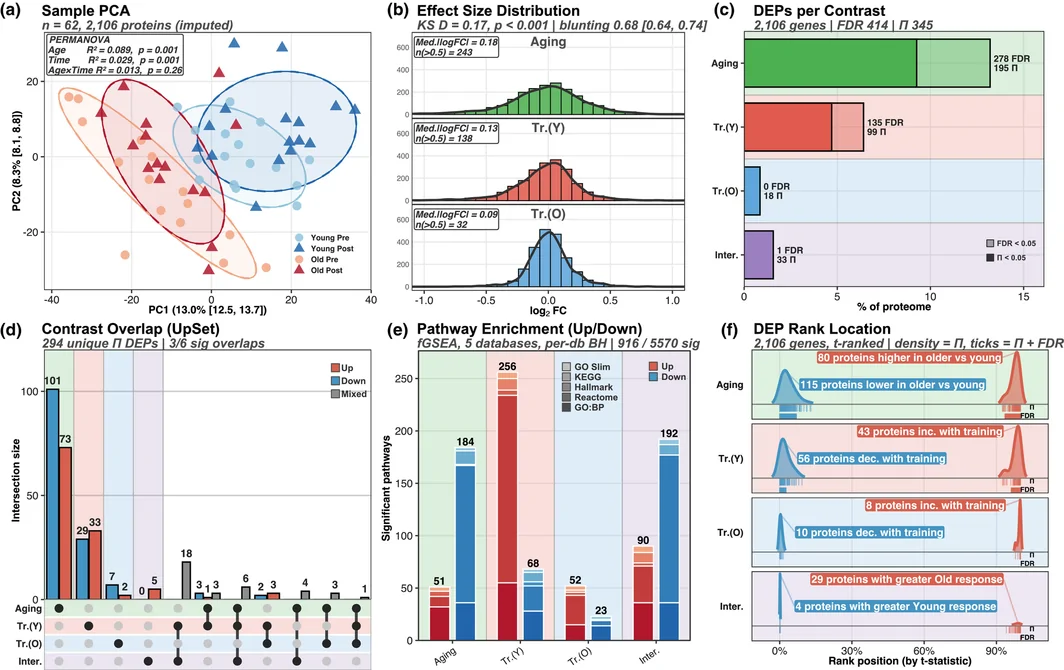
Proteome overview and differential expression landscape (2106 proteins, 62 samples). (a) PCA of the imputed protein matrix; PERMANOVA: Age *R* ^2^ = 0.089 (*p* = 0.001), Time *R* ^2^ = 0.029 (*p* = 0.001), Age × Time *R* ^2^ = 0.013 (*p* = 0.260). (b) Per‐contrast |log_2_FC| distributions showing progressive compression from Aging (median 0.18) to Training in Young (0.13) to Training in Old (0.09); Cliff's δ quantifies blunting of the older‐adult response relative to the younger. (c) DEP counts at the FDR and Π‐score thresholds. Nominal‐p counts are reported in the text and in Table S10 but are not plotted, so the panel cannot be read as three parallel results. (d) UpSet intersection of Π‐score DEPs across contrasts. (e) Fraction of fGSEA‐enriched pathways (BH‐corrected) across Hallmark, GO Slim, GO:BP, KEGG, and Reactome gene sets, separated by enrichment direction. (f) DEP rank location within the t‐statistic–ranked proteome per contrast. DEP, differentially expressed protein; FC, fold change; PCA, principal component analysis.

The central result is the gap between age groups at the false discovery rate: training altered 135 proteins at FDR <0.05 in younger adults and none in older adults, where the smallest adjusted *p*‐value was 0.156, shared by HPRT1 and FSCN1. The same asymmetry holds across all three thresholds (Figure 2c; diagnostics in Figure S4a, per‐protein abundance at each threshold in Figure S4b): the Aging contrast yielded the most DEPs at every threshold (195 at *Π* < 0.05; 278 at FDR <0.05; 570 at *p* < 0.05), followed by Training in Young (99; 135; 448), while Training in Old produced 18 at *Π* < 0.05 and 196 at *p* < 0.05. The 18 Π‐score hits are therefore proteins with large fold‐changes and nominal significance rather than proteins surviving multiplicity correction, and are reported with their adjusted *p*‐values in Table S10. The interaction contrast identified 33 DEPs (*Π* < 0.05), confirming that the attenuated older response is statistically robust (Figure 3d). Among the 195 aging‐significant proteins, the most strongly depleted in older muscle were sarcomeric components (LMOD2, log_2_FC = −2.35; ACTN3, −2.12), while the most elevated included GPT2 (+1.68, amino acid catabolism) and PLIN2 (+1.12, lipid droplet storage). Full per‐protein results for all contrasts are provided in Table S10, and the source data behind Figure 2 in Table S3.

**FIGURE 3:**
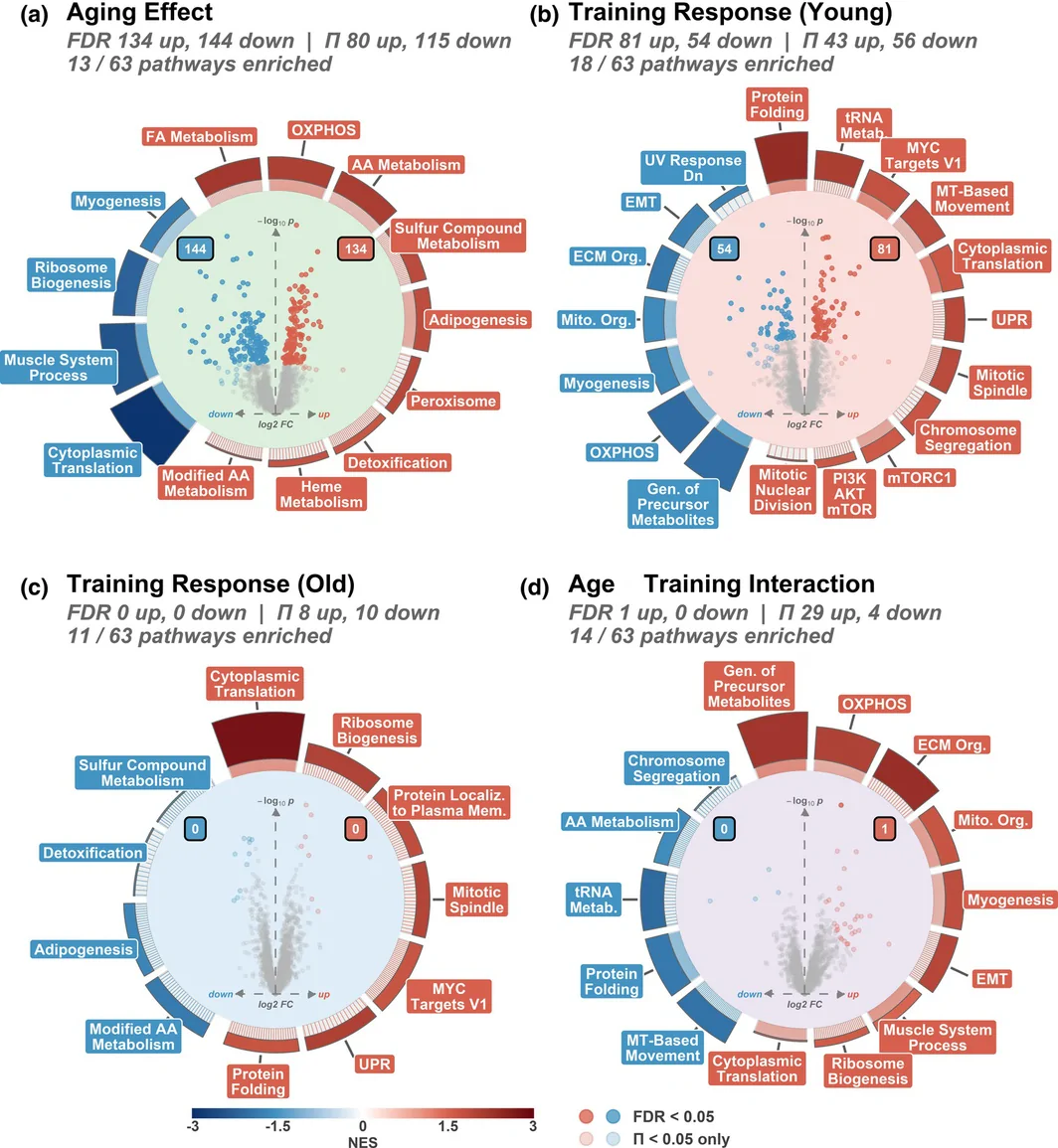
Volcano ring plots for the four primary contrasts. (a) Aging effect (Old_Pre − Young_Pre; 195 Π‐score DEPs; 235 enriched pathways). (b) Training in Young (Young_Post − Young_Pre; 99 DEPs; 324 pathways). (c) Training in Old (Old_Post − Old_Pre; 18 DEPs; 75 pathways). (d) Age × Training interaction ([Old_Post − Old_Pre] − [Young_Post − Young_Pre]; 33 DEPs; 282 pathways). Inner scatter plots show protein‐level log_2_FC vs. −log₁₀(*p*); red, upregulated; blue, downregulated. Outer ring insets display the 12 most significant pathways remaining after EnrichmentMap redundancy collapsing across the five gene‐set collections, colored by normalized enrichment score (NES); every representative is listed in Table S4. DEP significance threshold: *Π*‐score <0.05. DEP, differentially expressed protein; NES, normalized enrichment score.

UpSet intersection analysis of the 294 unique Π‐score DEPs revealed predominantly contrast‐specific signatures (Figure 2d), with Aging contributing the largest exclusive set (174 proteins, 59%), followed by Training in Young (62, 21%), and minimal higher‐order intersections. Protein set enrichment analysis (fGSEA; Hallmark, GO Slim, GO:BP, KEGG, and Reactome gene sets with BH correction per database) identified 916 significantly enriched pathway‐contrast combinations across all four contrasts (*p*adj <0.05; Figure 2e). The rank‐position distribution of Π‐score DEPs within the t‐statistic–ordered proteome confirmed that significant proteins cluster at the extremes of each contrast (Figure 2f). The Training Young and Interaction contrasts yielded the most pathway‐level signal (324 and 282 pathways, respectively), while Training in Old yielded 4.3‐fold fewer enriched pathways (75 vs. 324). Those 75 were nonetheless coherent: 52 were positively enriched, the strongest being translation initiation (NES = +2.85, *p*adj = 1.3 × 10^−14^), eukaryotic translation elongation (+2.83), the eIF2AK4/GCN2 amino‐acid‐deficiency response (+2.83), nonsense‐mediated decay (+2.79) and rRNA processing (+2.77). Because no individual protein survives FDR correction in this contrast, the older‐adult response is described at the pathway level, where coordinated shifts in ribosomal and translational machinery remain resolvable. Notably, the Interaction contrast—despite only 33 individual DEPs—identified substantial pathway‐level signal (282 pathways), indicating that age‐dependent training differences manifest as coordinated shifts across functionally related proteins.

### 3.3 Training responses are directionally conserved but magnitude‐compressed in older adults

To assess whether younger and older adults mount qualitatively similar or distinct training responses, we compared protein‐level changes between age groups. Flanked volcano plots revealed 99 Π‐score DEPs in younger versus 18 in older adults (Figure 3b,c). Despite this quantitative disparity, pathway‐level insets showed overlapping enrichment themes in both age groups, though younger adults exhibited pronounced oxidative phosphorylation suppression absent in older adults.

Training‐induced log_2_FC values were compared between age groups at two levels. Restricted to the 135 proteins reaching FDR <0.05 with training in younger adults, 110 (81.5%) changed in the same direction in older adults (Spearman *ρ* = 0.711). Across all 2094 proteins quantified in both age groups the same comparison gives 61.7% and *ρ* = 0.288 (*p* = 3.4 × 10^−41^; Figure 4a). Both are reported because the whole‐proteome figure is diluted by proteins with no resolvable effect in either group: sign concordance rose monotonically with effect magnitude, from 52.5% in the lowest quintile of min(|log_2_FC|) to 74.0% in the highest (trend *p* = 1.7 × 10^−12^; Figure S5a), and 599 of the 802 whole‐proteome sign disagreements (74.7%) involved a protein whose smaller effect fell below |log_2_FC| = 0.1. Per‐subject training deltas for the 135 proteins are shown in Figure S5b. The 25 proteins changing in opposite directions did not form a distinct biological class: 11 were Complex I subunits or mitochondrial import components, none reached FDR <0.10 in older adults, and their median |log_2_FC| in older adults was 0.038 against 0.151 for the 110 concordant proteins (*p* = 3.6 × 10^−8^), so the opposing sign is assigned to an effect indistinguishable from zero. The 110 concordant proteins were enriched for chaperonin‐mediated tubulin folding, cytoskeleton‐dependent intracellular transport and cytosolic tRNA aminoacylation (48 terms at FDR <0.05; Table S5). All six proteins reaching Π‐score significance in both age groups were directionally concordant. Effect sizes were systematically compressed in older adults (median |log_2_FC|: 0.13 vs. 0.09), consistent with an intact but attenuated training response (Figure 4c). Across the whole proteome the discordant set does group biologically: restricting to the 203 proteins with |log_2_FC| ≥ 0.1 in both age groups, over‐representation returns striated muscle contraction (14 of 28 measured, adjusted *p* = 2.1 × 10^−5^), intermediate‐filament‐based process (8 of 14, *p* = 0.017) and collagen fibril assembly (7 of 15, *p* = 0.026). The signal is one‐sided, belonging entirely to proteins that decrease with training in younger adults and increase in older adults (striated muscle contraction, adjusted *p* = 9.8 × 10^−8^). Tested on all 802 discordant proteins the leading terms are respiratory electron transport (63 of 104, adjusted *p* = 8.6 × 10^−4^) and the electron transport chain (53 of 82, *p* = 9.0 × 10^−4^).

**FIGURE 4:**
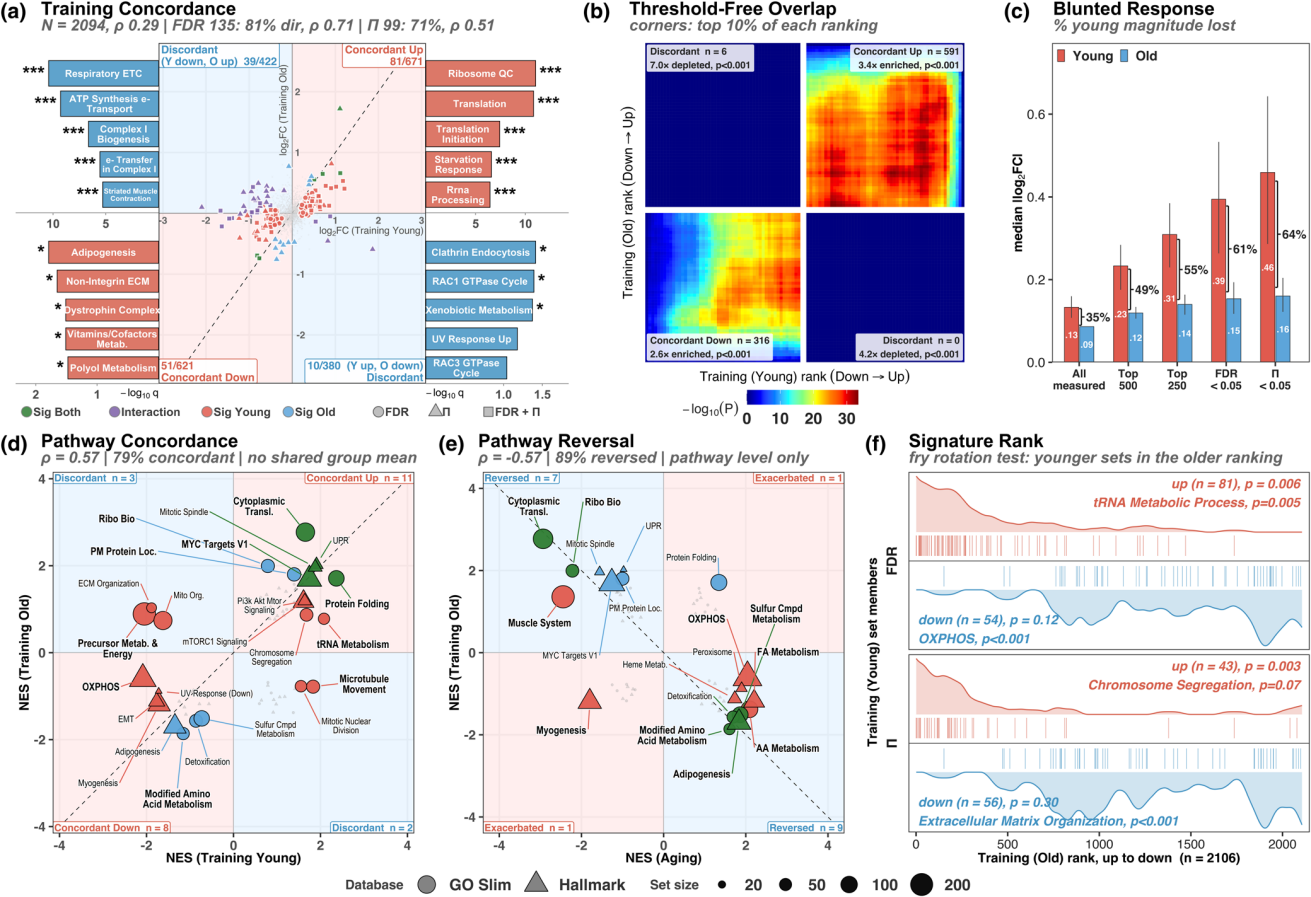
Training response concordance between younger and older adults. (a) Quadrant scatter of Training‐in‐Young vs. Training‐in‐Old log_2_FC (2094 proteins); 99 met *Π* < 0.05; 18 ORA‐enriched pathways at FDR < 0.05; Spearman *ρ* = 0.288 across all 2094 proteins (*p* = 3.4 × 10^−41^), rising to 0.711 across the 135 reaching FDR <0.05 with training in younger adults and 0.510 across the 99 reaching *Π* < 0.05. (b) Threshold‐free rank‐rank overlap of the two training contrasts; concordant quadrants are enriched and discordant quadrants depleted. (c) Median |log_2_FC| retained in older adults relative to younger, across nested protein sets from the whole proteome to the Π‐significant set. (d) Pathway‐level NES concordance across 63 a priori Hallmark/GO Slim sets; Spearman *ρ* = 0.571 (*p* = 1.65 × 10^−6^); 24 pathways significant, all of which survive the EnrichmentMap collapse that selects the labeled representatives. (e) Pathway‐level NES for Aging against Training‐in‐Old, the only level at which the reversal is reported; *ρ* = −0.57 with 89% of sets reversed. Both axes are measured from the same older‐adult baseline, one subtracting it and the other adding it, so some negative correlation appears in this panel even if training changed nothing; the panel is therefore descriptive, and the tests allowing for the shared baseline are in Figure S5a. (f) fry gene‐set rotation test of the younger‐adult training sets against the Training‐in‐Old ranking, drawing both significance criteria: At FDR <0.05, up *p* = 0.006 (*n* = 81) and down *p* = 0.12 (*n* = 54); at Π < 0.05, up *p* = 0.003 (*n* = 43) and down *p* = 0.30 (*n* = 56). FC, fold change; NES, normalized enrichment score; ORA, over‐representation analysis.

The rank‐rank map confirmed this pattern without reliance on arbitrary significance thresholds (Figure 4b), with strong concordance signals in both upregulated and downregulated quadrants and negligible discordant signal, indicating that the dominant pattern is concordance between age groups.

Pathway‐level concordance was stronger than protein‐level concordance (Spearman *ρ* = 0.571, *p* = 1.65 × 10^−6^; Figure 4d). Five pathways reached significance in both age groups, all directionally concordant. The largest age‐divergent signal was in oxidative phosphorylation (Hallmark; NES: −2.08 in young vs. −0.61 in old), with 24 of 63 curated Hallmark and GO Slim pathways reaching significance in at least one age group (concordance diagnostics in Figure S5a).

Among the 120 proteins reaching Π‐score significance in any training or interaction contrast (Figure 4a), 42 showed concordant upregulation, 38 concordant downregulation, and 40 discordant responses between age groups. Concordantly downregulated proteins were enriched for extracellular matrix organization, suggesting that ECM remodeling represents a shared prerequisite for training‐induced fiber adaptation regardless of age. A rotation gene set test (fry) confirmed that proteins upregulated by training in younger adults (*n* = 43 Π‐DEPs) responded concordantly in older adults (*p* = 0.003; Figure 4f), consistent with conservation of training direction despite magnitude compression.

### 3.4 Training in older adults opposes the aging signature

Having established that training responses are directionally concordant but magnitude‐compressed across age groups, we next examined how the older‐adult training response is oriented relative to the aging effect. Comparing the Aging contrast (195 Π‐score DEPs; Figure 3a) with the Training‐in‐Old contrast revealed 157 of 209 Π‐score DEPs with opposite‐sign changes between the Aging and Training‐in‐Old contrasts (75.1%) (Figure 3c). Pathway‐level ring insets showed a striking mirror image in that pathways downregulated with aging were upregulated with training, and vice versa. Per‐contrast ring and enrichment source data are in Table S4. Of the 209 proteins reaching Π‐score significance across the Aging or Training‐in‐Old contrasts, 157 (75.1%) fell in reversal quadrants. A threshold‐free rank‐rank map of the two contrasts shows the same asymmetry, densest in the Aging‐Down/Training‐Up quadrant, and is reported without a significance test (Table S5). Exacerbation signal was negligible. These are descriptive quadrant counts, and the Aging and Training‐in‐Old contrasts share the Old_Pre term with opposite signs, which makes their correlation negative before any biology enters. On the 278 proteins reaching FDR <0.05 for aging, the aging and older‐adult training coefficients correlate at *r* = −0.562, with 77.0% in opposite‐sign quadrants; on group‐mean axes with top‐n selection the observed value is *r* = −0.488. Against a null that preserves the coupling by swapping Pre and Post labels within each older participant and re‐selecting proteins, that value falls inside the null (null mean = −0.267, 95% CI −0.715 to 0.241, *p* = 0.25). Measuring the two axes in disjoint halves of the older participants gives *r* = −0.171 (95% CI −0.548 to 0.344). A protein‐permuted null tests only whether the correlation differs from zero and cannot address the shared baseline. Reversal classification was stable across |log_2_FC| thresholds (Table S5).

At the pathway level, 18 pathways showed significant enrichment in either or both contrasts (Figure 4e), with cytoplasmic translation showing the strongest reversal (NES: −2.93 with aging, +2.77 with training). Among the 30 highest‐magnitude aging DEPs, the majority showed directional reversal, enriched for muscle cell development and myogenesis, indicating that the structural proteins most affected by aging are precisely those most responsive to training‐mediated recovery. Taken together, the quadrant and pathway analyses describe a proteome in which the direction of the older‐adult training response tends to oppose the direction of the aging effect, most clearly in ribosomal and translational machinery and least in oxidative phosphorylation and lipid catabolism. Because the two contrasts share the older‐adult baseline, this is reported as a descriptive pattern rather than as evidence that training reverses the aging proteome.

### 3.5 Weighted co‐expression network analysis reveals age‐ and training‐associated protein modules

We constructed a signed WGCNA network from 2106 proteins across 62 samples, identifying 9 co‐expression modules (70–340 proteins; 1558 assigned, 548 unassigned; Figure 5a). Network construction used Pearson correlation with soft‐thresholding power 12, chosen on two criteria that agree: it is the lowest power reaching a scale‐free fit of *R* ^2^ = 0.877 against a cutoff of 0.87, relaxed from the conventional 0.90 per Langfelder & Horvath guidance for small‐sample studies, and it is the power the WGCNA FAQ recommends for a signed network with more than 40 samples (here 62). Requiring 0.90 would select power 13 and halve mean connectivity. Because paired pre/post samples violate the independence assumption of standard module‐trait correlations, associations were assessed via linear mixed models (eigengene ~ group + (1|subject)) with Kenward‐Roger degrees of freedom. Age was the dominant organizing principle, with three modules reaching BH‐corrected significance: Turquoise (340 proteins; Lipid Catabolism | TCA Cycle; *r* = +0.58, pBH = 2.5 × 10^−5^), Black (107; Glycolysis | Transl. Fact.; *r* = −0.60, pBH = 2.3 × 10^−5^), Yellow (210; Muscle Contraction | Sarcomere; *r* = −0.44, pBH = 0.008). The Blue module (272; chaperones/proteostasis) was the only module significantly associated with training in younger adults (*r* = +0.62, pBH = 0.002). No modules reached significance for Training in Old or the Interaction contrast. No stratified within‐group phenotypic association survived BH correction in either age stratum. The strongest was the change in the Green (Oxid. Phos. | Complex I) eigengene against the change in VL thickness in younger adults (*r* = +0.67, raw *p* = 0.006; pBH = 0.058 within that trait column, 0.94 across the full 180‐test screen). Among the baseline cross‐sectional correlations, baseline Yellow (muscle contraction) tracked baseline lean mass in the same stratum (*r* = −0.50, raw *p* = 0.047, *n* = 16, pBH = 0.47); both are reported as exploratory.

**FIGURE 5:**
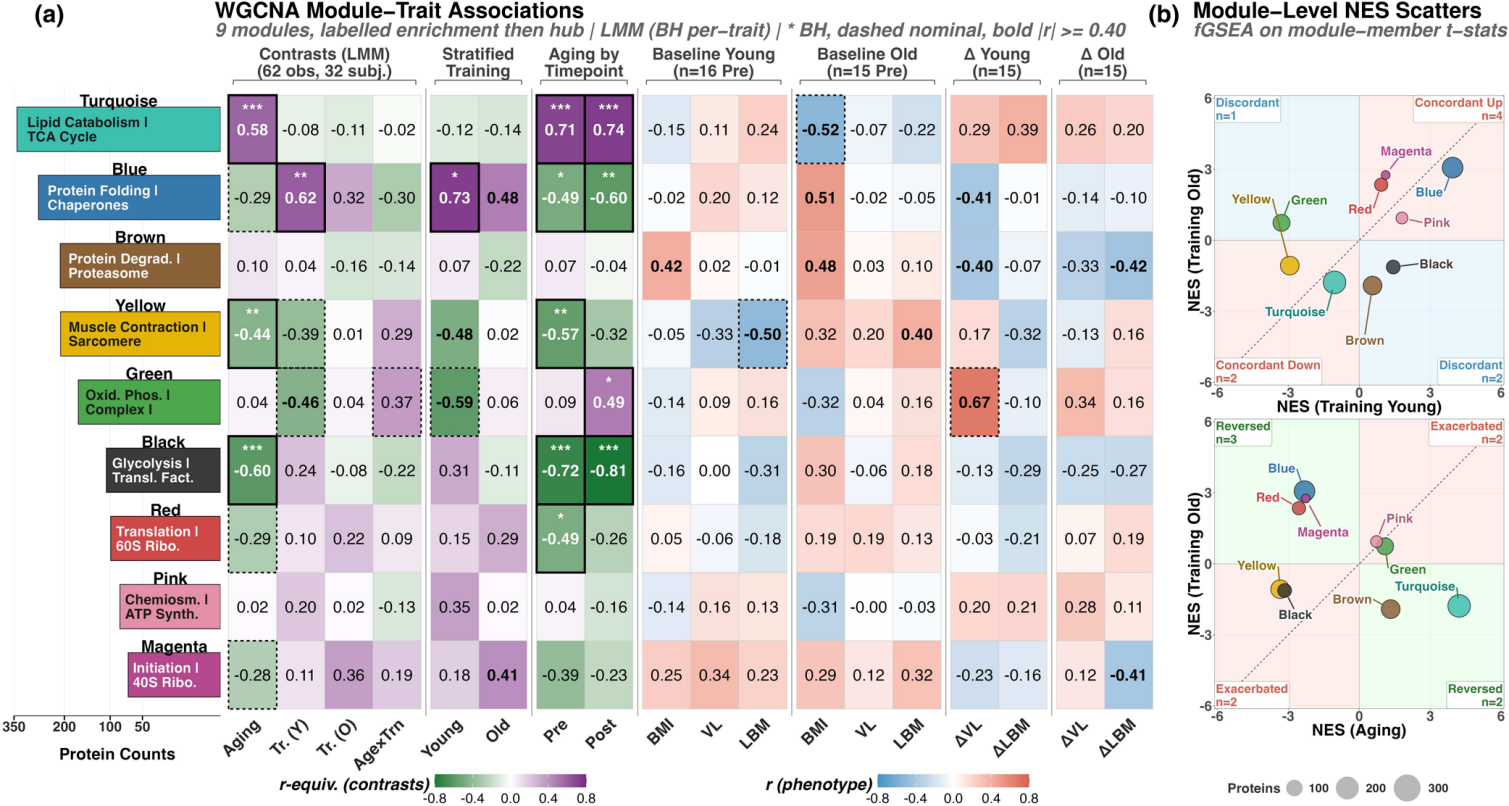
WGCNA co‐expression network analysis of 2106 proteins across 62 samples. (a) Module–trait association heatmap for nine modules. Left columns: r‐equivalents from paired linear mixed models (Aging, Training in Young, Training in Old, Age × Training); right columns: Stratified Pearson correlations with baseline phenotypes and training‐induced changes per age group. BH‐corrected significance and module sizes annotated. Three modules were age‐associated: Turquoise (Lipid Catabolism | TCA Cycle) (*r* = +0.58, p_BH = 2.5 × 10^−5^), Black (Glycolysis | Transl. Fact.) (*r* = −0.60, p_BH = 2.3 × 10^−5^), Yellow (Muscle Contraction | Sarcomere) (*r* = −0.44, p_BH = 0.008). (b) Module‐level NES scatters: Training‐in‐Young against Training‐in‐Old (upper) and Aging against Training‐in‐Old (lower). Bubble size reflects module protein count. NES, normalized enrichment score; WGCNA, weighted gene co‐expression network analysis.

Gene ontology enrichment of each module provided biological context (Table S6; network construction diagnostics in Figure S6). Per‐module *z*‐score heatmaps, eigengene trajectories, and over‐representation analysis (ORA) enrichment for the four age‐ and training‐associated modules are shown in Figure S8. Module names combine two derived halves: the largest protein family among the module's high‐connectivity core (members with kME ≥0.60 against their own eigengene) and the most significant specific GO biological process term, both tabulated per module in Table S6. The age‐elevated Turquoise module was dominated by organic acid catabolic processes (*p*adj = 6.7 × 10^−22^) and fatty acid catabolism. The age‐depleted Yellow module was enriched for striated muscle contraction (*p*adj = 2.0 × 10^−20^) and myogenesis. The age‐depleted Black module is the most weakly annotated of the nine: no GO biological process term reaches significance, the strongest being glucose catabolism at *p*adj = 0.083, though KEGG glycolysis (*p*adj = 1.2 × 10^−4^) and Hallmark hypoxia (*p*adj = 3.8 × 10^−3^) do. Its high‐connectivity core is four translation elongation and initiation factors and two proteasome lid subunits, with no glycolytic protein clearing the core threshold. The training‐responsive Blue module was enriched for protein‐folding processes including protein folding (*p*adj = 3.4 × 10^−7^) and the Hsp90 chaperone cycle (*p*adj = 9.0 × 10^−7^). Brown showed enrichment for proteasomal degradation (*p*adj = 1.1 × 10^−4^) and gluconeogenesis (*p*adj = 1.1 × 10^−4^). Green was dominated by ATP synthesis‐coupled electron transport (*p*adj = 9.9 × 10^−56^). Magenta was enriched for translation initiation and Pink for ATP synthase and chemiosmotic coupling.

### 3.6 Baseline proteomic profiles discriminate age group

To determine which co‐expression modules carry outcome‐relevant signal, we performed univariate module‐by‐outcome discrimination using module eigengenes across the annotated WGCNA modules and six outcomes (Figure S7), of which the age contrast is shown in Figure 6a and the pre‐to‐post contrast in Figure 6b. The strongest signals were age‐discriminating. Black/M6 (Glycolysis | Transl. Fact.) separated Young from Old completely (AUC = 1.00, permutation *p* < 0.001, *q* < 0.010), followed by Turquoise/M1 (Lipid Catabolism | TCA Cycle; AUC = 0.96, *q* < 0.010), Blue/M2 (Protein Folding | Chaperones; AUC = 0.90, *q* < 0.010) and Yellow/M4 (Muscle Contraction | Sarcomere; AUC = 0.80, *q* = 0.032). Black's complete separation should be read against the stability caveat below. Within‐subject Pre‐to‐Post timing was weaker across all modules (AUCs 0.47–0.86 across the pooled and age‐stratified comparisons; 0.47–0.73 pooled), and no single module reached BH significance for ΔVL or ΔLBM responder status. Per‐module ROC curves across all outcomes are shown in Figure S7, with the per‐module values in Table S7.

**FIGURE 6:**
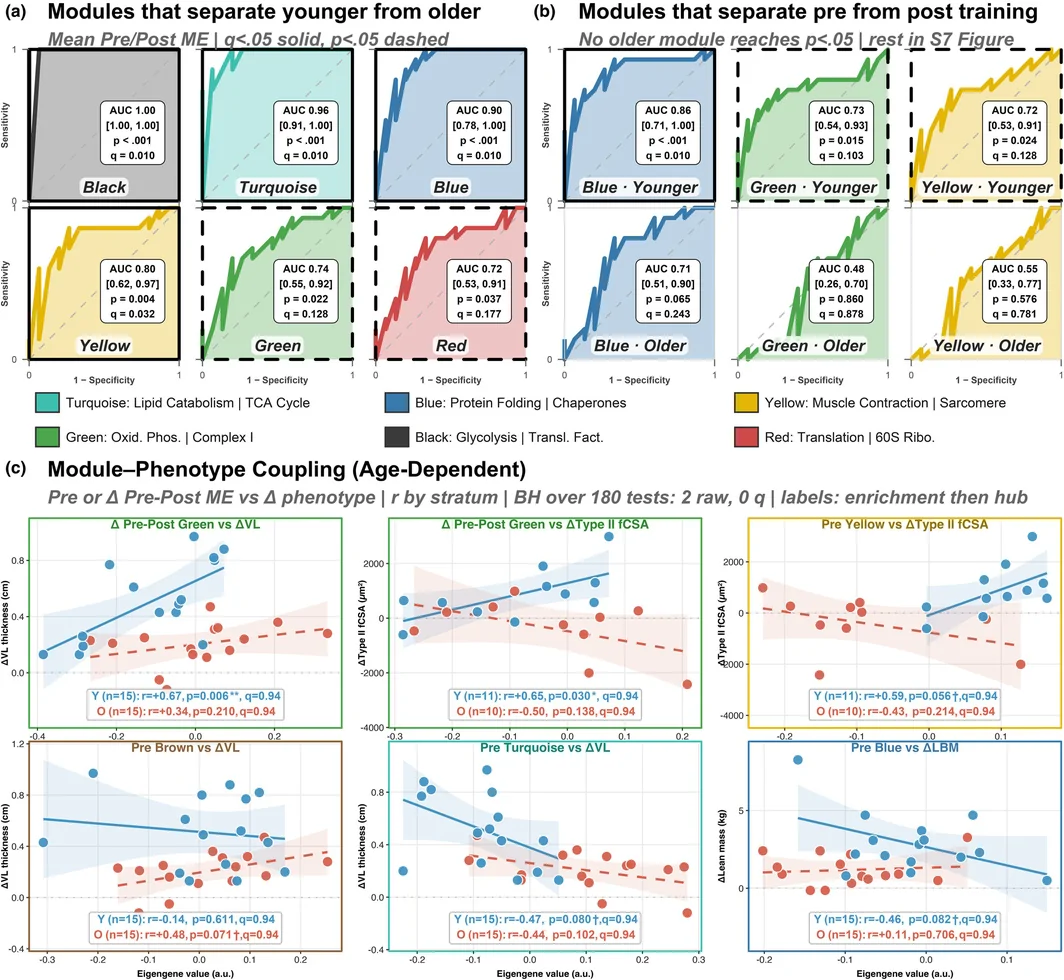
Module eigengene discrimination and age‐stratified phenotype coupling. (a) Univariate ROCs for the module eigengenes that separate younger from older adults. Thick solid border: BH *q* < 0.05; thick dashed: Nominal *p* < 0.05. Black (Glycolysis | Transl. Fact.) (AUC = 1.00) and Turquoise (Lipid Catabolism | TCA Cycle) (AUC = 0.96) were the strongest age discriminators (both *q* < 0.010); Black's module composition is not robustly recovered under leave‐one‐subject‐out refitting, so its separation is reported with that caveat. (b) The same ROCs for the pre‐ versus post‐training contrast, each module drawn in both age strata; no older‐adult module reaches *p* < 0.05, and the full grid across all six outcomes is in Figure S7. (c) The six module–phenotype cells with the lowest raw p across the 180‐test screen, each drawn for both age strata. Predictors are module eigengene values, Δ or baseline; outcomes are training‐induced changes in VL thickness, lean body mass and Type II fiber cross‐sectional area. Every cell prints the stratified Pearson *r*, its raw p and its BH‐corrected *q*; *q* = 0.94 throughout, so none of these associations survives correction across the screen. AUC, area under the curve; fCSA, fiber cross‐sectional area; LBM, lean body mass; ROC, receiver operating characteristic; VL, vastus lateralis.

Leave‐one‐subject‐out validation confirmed that these in‐sample AUCs were not inflated by optimism bias: both fixed‐module projection LOSO and full WGCNA‐refit LOSO reproduced the Turquoise (0.96/0.96), Blue (0.92/0.93), and Yellow (0.80/0.81) out‐of‐fold AUCs within narrow confidence intervals and returned 1.00 for Black by both routes, the refit value resting on six folds (Figure S7). Within‐subject change (ΔME) did not discriminate age (AUC = 0.48, *p* = 0.91), whereas the Pre + Post + ΔME combined stack reached AUC = 0.96 (*p* = 0.017), supporting the view that age signal lives in static, cross‐subject proteome state. Raw‐protein (k = 10) Age classifiers achieved AUC = 0.991 (perm *p* < 0.005), whereas phenotype‐only discrimination was not significant (AUC = 0.78, *p* = 0.17), and Brown and Black were the least reproducible modules under leave‐one‐subject‐out network refitting, whereas the seven remaining modules — including the age‐discriminating Turquoise, Blue, Yellow, Red, and Green—reproduced reliably. The strong Black/M6 age‐discrimination AUC should therefore be interpreted with the caveat that this module's protein composition is not robustly recovered on *n* − 1 subsamples.

Module eigengenes also tracked phenotypic adaptation with clear age stratification (Figure 6c). In younger adults the change in the Oxidative Phosphorylation eigengene tracked the change in VL thickness (*r* = +0.67, raw *p* = 0.006), while cross‐sectionally baseline Muscle Contraction tracked baseline lean mass (*r* = −0.50, raw *p* = 0.047, *n* = 16). Older adults showed weaker or null associations. The module‐phenotype associations were drawn from a 180‐test screen (9 modules × 2 predictors × 5 outcome changes × 2 age strata); the baseline cross‐sectional correlations form a separate per‐trait family of 10 modules. None survived BH correction in either family, and all should be interpreted as exploratory age‐dependent patterns.

Within the age‐associated Turquoise module (340 proteins), 10 hub candidates were identified by module membership (kME >0.75), representing proteins central to the co‐expression network. The top hub proteins (ETFB, IDH2, ALDH5A1, ETFA, CRAT) are dominated by fatty acid and organic acid catabolic enzymes, reinforcing the metabolic identity of this age‐associated module. These are the protein‐level features that distinguish aging muscle from younger muscle. Whether they are also the features limiting its capacity for training‐induced growth is not answered by these data: no module‐phenotype association survived correction.

## 4 DISCUSSION

The present study leveraged deep quantitative proteomics to characterize how aging modulates the skeletal muscle response to resistance training. Five principal findings emerged. First, despite both age groups implementing a similar 10–12‐week program, older adults exhibited markedly attenuated proteome remodeling with 5.5‐fold fewer training‐responsive proteins and zero FDR‐significant proteins. Second, despite this compression, 81.5% of the training‐responsive proteins identified in younger adults changed in the same direction in older adults, indicating a conserved but amplitude‐limited training response in older individuals. Third, training‐induced changes in older adults ran opposite to the aging effect, most clearly in ribosomal and translational machinery. Fourth, co‐expression network analysis identified age‐divergent biological programs, with oxidative phosphorylation emerging as the pathway most resistant to training‐mediated reversal and most divergent between age groups. Finally, baseline proteomic profiles discriminated age group, while no module‐phenotype association reached significance after correction. Collectively, these results suggest that aging constrains the amplitude of proteome remodeling but does not render the underlying adaptive programs immutable, and they leave open whether baseline proteomic state sets the boundaries of response capacity, while training can partially restore individual protein abundances within those boundaries. These findings will be expanded upon in the paragraphs below.

### 4.1 Attenuated but conserved training responses across age

The magnitude of the training response deficit in older adults is substantial. At the Π‐score threshold, younger adults produced 99 DEPs compared with 18 in older adults—5.5‐fold attenuation (Figure 2c). At FDR <0.05 the disparity was absolute (135 vs. 0), and relaxing the threshold to FDR <0.10 does not change it: the smallest adjusted *p*‐value anywhere in the older‐adult training contrast is 0.156 (HPRT1). The 18 proteins passing the Π‐score threshold should therefore be read as the largest and most nominally significant effects within a contrast that has no FDR‐significant findings, not as an alternative list of confirmed responders. We accordingly base the older‐adult training narrative on pathway‐level evidence, where 75 gene sets reached significance and ribosomal and translational machinery was coordinately upregulated (NES up to +2.85, *p*adj = 1.3 × 10^−14^), while noting that no co‐expression module reached significance for training in older adults. Composite metrics such as the Π‐score remain useful for ranking effects in underpowered contrasts, but they do not substitute for multiplicity control. The consistent pattern across all significance thresholds supports genuine biological attenuation, consistent with O'Leary et al. (2025), who reported zero FDR‐significant proteins in either age group despite detecting significant pathways, and our past proteomic work showing that aging predominantly affects the non‐myofibrillar protein pool with marginal training‐induced changes (Roberts et al., 2024). Despite this quantitative deficit, the training response was not qualitatively altered. Protein‐level concordance was 81.5% (*ρ* = 0.711) among the 135 proteins that responded to training in younger adults at FDR <0.05, and 61.7% (*ρ* = 0.288, *p* = 3.4 × 10^−41^; Figure 4a) across the whole proteome; the gap is explained by proteins whose effect is too small for its sign to be informative rather than by divergent biology. The rank‐rank map confirmed strong concordance with negligible discordant signal (Figure 4b). This “conservation with compression” pattern suggests that therapeutic strategies aimed at amplifying existing responses, rather than redirecting their trajectory, may hold promise for long‐term muscular development in older adults. This is further supported by Lee‐Odegard et al. (2025), who showed that proteomic aging (ProtAgeGap) was inversely associated with physical activity in 45,438 UK Biobank participants, with a 12‐week exercise intervention decreasing ProtAgeGap by approximately 10 months.

Pathway‐level concordance (*ρ* = 0.571, *p* = 1.65 × 10^−6^) substantially exceeded protein‐level concordance, suggesting that coordinated functional programs are better preserved across age than individual protein effect sizes, with 24 of 63 curated Hallmark and GO Slim pathways reaching significance in at least one age group (Figure 4d). The largest age‐divergent signal was in oxidative phosphorylation (Hallmark; NES: −2.08 in young vs. −0.61 in old). This extends proteomic observations from transcriptomic precedents such as those by Raue et al. (2012) who reported seven‐fold fewer responsive transcripts in older adults, and Thalacker‐Mercer et al. (2013) who reported that baseline transcriptomic profiles predict response heterogeneity. Hence, individual protein effects may be buffered by functional overlap within coordinated pathways, consistent with multi‐omic exercise studies (MoTr, Lead et al., 2024). In our data, aging and training shared minimal protein‐level overlap (85% of *Π*‐score DEPs were contrast‐exclusive), yet pathway concordance between age groups (*ρ* = 0.571) nearly doubled protein concordance (*ρ* = 0.288). Despite age explaining modest global variance (PERMANOVA *R* ^2^ = 8.9%), 195 DEPs were identified, indicating that aging remodels the proteome through many small, coordinated shifts best captured at the pathway and network level. Notably, Michie et al. (2024) reported that older adults exhibit chronically activated unfolded protein response (UPR) pathways at rest, while the adaptive UPR response to acute exercise is similarly activated across age, consistent with the concordant UPR upregulation we observed in both age groups, further underscoring the proteostatic disruptions that characterize aging muscle.

### 4.2 Resistance training partially reverses the aging proteome

Training‐induced changes in older adults tend to run opposite to the direction of the aging effect, though the two contrasts are not independent. Among 209 proteins meeting the Π < 0.05 threshold across the Aging and Training‐in‐Old contrasts, 157 (75.1%) showed opposite‐sign changes, and among the top 30 aging‐affected DEPs, 22 (73%) showed directional reversal. A rank‐rank map of the two contrasts shows the same asymmetry, densest in the Aging‐Down/Training‐Up quadrant (Table S5), extending the transcriptomic reversal reported by Melov et al. (2007) to the proteome level. The strongest reversal occurred in ribosomal and translational machinery, with cytoplasmic translation showing complete directional inversion (Aging NES = −2.93; Training NES = +2.77; Figure 4e). This is particularly notable given evidence that ribosome biogenesis capacity determines hypertrophic outcomes in older adults, as mentioned previously. In contrast, oxidative phosphorylation showed minimal reversal, indicating a hierarchy in which translational programs are more responsive to exercise‐mediated restoration than mitochondrial metabolic programs. These directional counts are descriptive. The aging contrast and the training‐in‐older‐adults contrast both contain Old_Pre, with opposite signs, so a negative correlation between them is expected on structure alone. Once that coupling is preserved in the null, the correlation is no longer distinguishable from it (observed *r* = −0.488 against a null mean of −0.267, *p* = 0.25), and a split‐half estimate measuring the two axes in disjoint older participants gives *r* = −0.171 with a confidence interval spanning zero (Figure S5a). We therefore treat the reversal pattern as hypothesis‐generating rather than established and note that separating it from the shared baseline requires defining the aging signature in participants other than those in whom the training response is measured.

That 75.1% of aging‐affected proteins show opposite‐sign training responses, including 73% of the most strongly affected, would suggest that a substantial portion of age‐related proteomic change reflects modifiable homeostatic shifts rather than irreversible degeneration, if the two contrasts were independent. They are not, and the coupling‐aware estimates above do not support that reading on the present data. This is consistent with Hulmi et al. (2025), who demonstrated that RT‐induced proteomic changes are partially reversible upon detraining, indicating that skeletal muscle retains inherent proteome plasticity. Complementary evidence at the epigenomic level further supports this capacity, as DNA methylation signatures of prior hypertrophic stimuli persist through detraining and may prime subsequent retraining responses (Seaborne et al., 2018).

### 4.3 Co‐expression network architecture

WGCNA identified nine co‐expression modules organizing 1558 of 2106 proteins (548 unassigned), and the module‐level picture is the compression seen per protein and per pathway, one level of aggregation up. Age organized the network: Turquoise (Lipid Catabolism | TCA Cycle; *r* = +0.58, pBH = 2.5 × 10^−5^), Black (Glycolysis | Transl. Fact.; *r* = −0.60, pBH = 2.3 × 10^−5^) and Yellow (Muscle Contraction | Sarcomere; *r* = −0.44, pBH = 0.008) each separated the age groups under linear mixed models accounting for the paired design. Read together they describe one shift rather than three separate findings: fuel oxidation up, translational machinery down, contractile apparatus down. Training reached one module only, Blue (Protein Folding | Chaperones; *r* = +0.62, pBH = 0.002), and only in younger adults; no module reached significance for training in older adults or for the interaction. Module‐level enrichment showed the same two patterns at coarser grain (Figure 5b). Training enrichment diverged between age groups: only Blue (chaperones and protein folding) reached significance in both younger and older adults (NES +3.95 and + 3.07), with four modules significant in younger adults only and four in older adults only. Against the aging contrast the pattern inverted: all five modules significant in both Aging and Training‐in‐Old carried opposite signs, Turquoise and Brown rising with age and falling with training and Blue, Magenta and Red the reverse. As at the protein and pathway levels these are descriptive, because the two contrasts share the Old_Pre term and are not independent. The same ordering therefore holds at every level of aggregation examined—a large aging signal, a smaller training signal in younger adults, and an older‐adult training signal the data cannot resolve—while the architecture itself stays put: eight of nine modules were strongly preserved between pre‐ and post‐training networks (*Z* > 10), with Magenta moderately preserved (*Z* = 8.1). The co‐expression architecture of the skeletal muscle proteome is thus largely stable across the intervention even as individual protein abundances shift. This parallels the WGCNA‐derived transcriptional networks of Lavin et al. (2021), who showed that baseline molecular network state shapes hypertrophic response heterogeneity in older adults undergoing 14 weeks of RT. Within that stable frame, Green (Oxid. Phos. | Complex I) carries the one outcome‐adjacent signal: its eigengene change tracked VL hypertrophy in younger adults (*r* = +0.67, raw *p* = 0.006) without surviving correction within that stratum (pBH = 0.058), so it is reported as exploratory and no direction of effect is read into it. The reproducible age signal is carried by Turquoise and Yellow. Black returns the largest age correlation (*r* = −0.60, pBH = 2.3 × 10^−5^) but is the least reproducible module in the network, so it is read as consistent with the translational shift rather than as the evidence for it, with Red and Magenta falling in the same direction without reaching significance (pBH = 0.11 and 0.13); at the pathway level the six most strongly age‐depleted gene sets are all ribosomal or translational (NES −3.10 to −2.94). The agreement is directional rather than compositional—Black contributes 8 of cytoplasmic translation's 93 leading‐edge proteins against 37 from Red and 27 from Magenta—but both levels place translational machinery at the center of the aging signal. Brown and Black were the least reproducible modules under leave‐one‐subject‐out refitting, so their composition, and any biology read from them, should be treated as provisional.

### 4.4 Mitochondrial and metabolic pathway remodeling

Younger adults showed strong suppression of oxidative phosphorylation following training (Hallmark OxPhos NES = −2.08), which may reflect a shift in biosynthetic resources toward growth‐related programs, while this response was almost entirely absent in older adults (NES = −0.61), generating the strongest interaction effect among all pathways (NES = +2.02). Aging itself was associated with elevated oxidative phosphorylation gene products (NES = +2.04), which may represent compensatory upregulation in response to declining mitochondrial efficiency. Robinson et al. (2017) reported that resistance training increased mitochondrial protein synthesis in older but not younger adults, suggesting older muscle responds to energy demands by augmenting mitochondrial biogenesis rather than undergoing the glycolytic shift observed in younger adults, though Jessen et al. (2026) recently showed that RT‐induced OxPhos suppression occurs predominantly in type I fibers, suggesting the whole‐muscle signal reflects fiber‐type‐specific remodeling. This age‐specific failure to remodel mitochondrial protein content may reflect impaired mitochondrial quality control, as mitochondrial dysfunction is increasingly recognized as both a hallmark and potential driver of biological aging. The MoTrPAC mitochondrial atlas further demonstrates tissue‐specific mitochondrial multi‐omic remodeling during endurance training (Amar et al., 2024), with skeletal muscle showing an absence of a coordinated oxidative protein upregulation in our RT context.

### 4.5 Baseline proteomic signatures discriminate age

Module eigengenes decomposed age‐ and training‐related signal across distinct biological programs. Univariate per‐module ROCs indicated that age separation is carried primarily by metabolism‐linked modules (Turquoise (Lipid Catabolism | TCA Cycle), AUC = 0.96, *q* < 0.010) and proteostasis (Blue (Protein Folding | Chaperones), AUC = 0.90, *q* < 0.010; Figure 6a), with Yellow (Muscle Contraction | Sarcomere) providing a weaker but significant signal (AUC = 0.80, *q* = 0.032), while within‐subject training‐time discrimination was weaker across all modules (pooled Pre vs. Post AUCs 0.47–0.73). Module eigengenes also tracked phenotypic adaptation with clear age stratification (Figure 6c): in younger adults the change in the Oxidative Phosphorylation eigengene tracked the change in VL thickness (*r* = +0.67, raw *p* = 0.006), while cross‐sectionally baseline Muscle Contraction tracked baseline lean mass (*r* = −0.50, raw *p* = 0.047, *n* = 16), and older adults showed weaker or null associations.

### 4.6 Mechanistic and clinical implications

The convergence of multiple independent analytical approaches—differential expression, concordance, and network analysis—on common biological themes provides insight into how aging can affect the proteome. The pronounced attenuation at the protein level, combined with preserved pathway‐level concordance, suggests that aging introduces bottlenecks at transcriptional and post‐transcriptional levels that compress individual protein effect sizes while preserving functional program coordination. Hence, we posit that the anabolic signaling infrastructure necessary for maximal proteome remodeling is reduced with aging, though the present design cannot localize that constraint to a specific signaling step. If the conservation‐with‐compression pattern holds, it would favor therapeutic strategies that amplify existing response amplitudes over those that redirect response trajectories. The ribosome biogenesis pathway emerges as a particularly attractive intervention target given that ribosomal proteins were among the most strongly restored by training in older adults (Figure 4e), and ribosome biogenesis capacity predicts hypertrophic outcomes in older adults (Stec et al., 2016); though the relative importance of translational capacity versus efficiency may shift as training progresses, with greater hypertrophy linked to lower ribosome‐related gene expression after prolonged RT (Brown et al., 2024). While speculative, pharmacological, nutritional, or other interventional strategies aimed at enhancing both ribosome biogenesis and efficiency—such as leucine supplementation or resistance exercise combined with essential amino acid provision—may augment the amplitude of proteome remodeling in older adults.

### 4.7 Limitations

First, the analysis is secondary, drawing from parent RT investigations with shared training protocols but different nutritional supplementation paradigms (peanut protein and beetroot juice), and participant‐level random intercepts accounted for within‐subject correlation. Supplementation is perfectly nested within age group in this pooled sample, so a supplement term is confounded with the age term and was not fitted. The baseline stratum difference is therefore not separable from the cohort difference between parent studies, while the training response reported here does not appear to depend on supplementation. Figure S9 shows the cohort structure and every adjusted fit. Second, the sample sizes (*n* = 17 younger, *n* = 15 older) provided adequate power for detecting moderate‐to‐large training effects in younger adults but limited power for the interaction contrast. The complete absence of FDR‐significant findings in Training Old likely reflects both biological attenuation and power limitations. Third, older adults carried substantially more fat mass and a higher fat‐to‐lean ratio at baseline, and neither changed differentially with training. Adiposity therefore cannot account for the difference in training response, which is measured within subjects, but it is not separable from chronological age in the cross‐sectional aging contrast and has not been adjusted for. Fourth, the aging component is cross‐sectional, and comparisons between age groups may reflect cohort differences rather than biological aging per se, and a single post‐training timepoint cannot capture the kinetics of proteome remodeling. Fifth, all participants were male, and results may not generalize to females given known sex differences in skeletal muscle physiology and training adaptations. Additionally, all biopsies were obtained from the VL, and results may not generalize to muscles with different fiber‐type compositions or functional demands, particularly upper‐body or predominantly slow‐twitch muscles such as the soleus. Age‐related impairments in recovery from exercise‐induced muscle damage (Li et al., 2024) may also produce differential adaptive responses depending on the RT protocol employed, and results should be interpreted within the context of the specific training design used herein. Finally, the older cohort (mean age ~58 years) is younger than many participants sampled in aging studies across the literature, and results may underestimate effects in adults over 65 years old.

### 4.8 Conclusions

In summary, these data provide evidence that aging markedly attenuates but does not qualitatively alter skeletal muscle proteome plasticity in response to resistance training. The training response in older adults, while compressed in magnitude, retains directional conservation with younger adults. It also runs opposite to the aging effect for a substantial fraction of proteins, most clearly in ribosomal and translational machinery and least in mitochondrial metabolic programs, but the aging and older‐adult training contrasts share a baseline, and coupling‐aware tests do not separate that pattern from the structure it inherits. Whether training reverses the aging proteome remains open. Direct proteomic comparisons of resistance training across age groups remain scarce, and resolving whether training reverses the aging proteome will require defining the aging signature in participants other than those in whom the training response is measured.

## AUTHOR CONTRIBUTIONS

**Dustyn T. Lewis:** Conceptualization; formal analysis; investigation; methodology. **J. Max Michel:** Investigation; methodology. **Mason C. McIntosh:** Investigation; methodology. **Dakota R. Tiede:** Investigation; methodology. **Daniel L. Plotkin:** Investigation; methodology. **Madison L. Mattingly:** Investigation; methodology. **Nicholas J. Kontos:** Investigation; methodology. **George Kontos Jr.:** Investigation; methodology. **Breanna J. Mueller:** Investigation; methodology. **Samuel C. Norton:** Investigation; methodology. **Joshua S. Godwin:** Investigation; methodology. **Brad J. Schoenfeld:** Formal analysis; resources. **Melissa D. Boersma:** Methodology; resources; software; validation. **Andrew D. Frugé:** Funding acquisition; project administration. **C. Brooks Mobley:** Project administration; supervision. **Andreas N. Kavazis:** Project administration; supervision. **Michael D. Roberts:** Conceptualization; funding acquisition; project administration; resources; supervision.

## FUNDING INFORMATION

Funding for the three original investigations can be found in parent publications. Proteomics analysis was funded by discretionary laboratory funds of MDR. DTL, DLP, and SCN were fully supported by Auburn University Presidential Fellowships. DRT was partially supported by an Auburn University College of Education Dean's Fellowship. MCM was fully supported by the American Physiological Society (Porter Fellowship).

## CONFLICT OF INTEREST STATEMENT

The authors declare no conflicts of interest.

## ETHICS STATEMENT

Human studies were approved by the Auburn University Institutional Review Board (Protocol numbers 19‐249, 20‐136, & 24‐863) and conducted in accordance with the Declaration of Helsinki.

## CONSENT

Written informed consent was obtained from all individual participants included in the original studies.

## Supporting information


**Table S1.** Pipeline QC source data—F00 panel‐level data covering normalization (panels A–G) and imputation (panels H–N) diagnostics.

**Table S2.** Figure 1 source data—phenotypic responses to resistance training (training volume, lean body mass, VL thickness, baseline characteristics108) and Figure S2 panel data.

**Table S3.** Figure 2 source data—proteome overview and DEP landscape (PCA, log_2_FC density, DEP counts, UpSet intersections, fGSEA, rank barcode) and Figure S3 panel data.

**Table S4.** Figure 3 source data—volcano ring plots, per‐contrast pathway enrichment, and Figure S4a/S4b panel data (model diagnostics/protein groupings at each significance threshold).

**Table S5.** Figure 4 source data—training concordance between younger and older adults (ORA quadrants, rank‐rank overlap, magnitude retention, NES scatters and the fry barcode) and Figure S5a panel data.

**Table S6.** Figure 5 source data—WGCNA module assignments, hub proteins (kME ≥Q90), module enrichment, module‐trait associations (LMM with Kenward‐Roger df, BH‐corrected), module‐level NES summaries.

**Table S7.** Figure 6 source data—univariate per‐module ROCs, leave‐one‐subject‐out validation, and age‐stratified module‐phenotype coupling.

**Table S8.** Per‐sample metadata, baseline phenotype, cyclic‐loess–normalized log_2_ protein matrix (2106 proteins × 62 samples), and per‐protein/per‐sample missingness summaries.

**Table S9.** Post‐imputation protein matrix (missForest; OOB NRMSE = 0.108) with Boolean imputation mask and per‐protein MAR vs. MNAR classification (3‐method consensus). Includes 16‐method imputation benchmark composite ranking.

**Table S10.** Differential expression results across four contrasts with Π‐score (per Xiao et al. 2014), FDR, and post hoc power analysis.

**Figure S1a.** Quality‐control and normalization pipeline. (A) Protein filter cascade (2837 to 2106). (B) Per‐protein missingness. (C) Pre‐normalization PCA. (D) Post‐normalization PCA. (E) Eta‐squared variance decomposition. (F) Outlier consensus scoring. (G) Per‐sample missingness.

**Figure S1b.** Imputation diagnostics. (H) Missingness classification scatter (MAR vs. MNAR). (I) Missingness classification counts. (J) 16‐method imputation benchmark (OOB NRMSE = 0.108). (K) Imputation density overlay. (L) MNAR shift diagnostic. (M) Sample integrity post‐imputation. (N) DEP sensitivity heatmap.

**Figure S2.** Phenotypic responses to resistance training by age group. (A) Deadlift one‐repetition maximum. (B) Type II fiber cross‐sectional area. (C) Type I fiber cross‐sectional area. (D) Whole‐body DXA fat mass. (E) Fat‐to‐lean mass ratio.

**Figure S3.** Proteome‐level coefficient of variation by experimental group. (A) Per‐protein variability (CV%). (B) Inter‐individual variability (CV%). (C) Intra‐individual proteomic variability across the imputed matrix.

**Figure S4a.** Differential expression model diagnostics across contrasts. (A) *p*‐value histograms. (B) Pi‐score distributions. (C) FDR distributions. (D) MA plots. (E) DEP retention after outlier removal.

**Figure S4b.** Per‐protein abundance across the two significance thresholds. Row *z*‐scored abundance per sample, hierarchically clustered within functional groups (GO Slim and Hallmark). (A) Aging at FDR <0.05. (B) Aging at *Π* <0.05. (C) Training‐in‐Young at FDR <0.05. (D) Training‐in‐Young at *Π* <0.05.

**Figure S5a.** Training concordance robustness diagnostics. (A) Spearman rho bootstrap. (B) GO Slim category distribution by quadrant. (C) Sign concordance across quintiles of the smaller absolute log_2_ fold change. (D) Shared‐baseline coupling diagnostic for the Aging and Training‐in‐Old correlation.

**Figure S5b.** Protein‐level training response across age groups. Per‐subject log_2_ change (Post − Pre) for the proteins reaching FDR <0.05 with training in younger adults, across the paired younger and older participants. Rows are grouped by the direction of the younger‐adult response and annotated with whether the older‐adult response shares that sign; gray cells are proteins not quantified in that participant. The right‐hand panel gives the most enriched over‐representation terms for the increased and for the decreased sets.

**Figure S6.** WGCNA network construction diagnostics. (A) Scale‐free topology fit. (B) Protein dendrogram with module assignments. (C) Subcellular compartment enrichment. (D) Pearson vs. biweight midcorrelation module overlap.

**Figure S7.** Module eigengene discrimination and module–phenotype association across the full sweep. (A) Per‐module ROC grid: one univariate AUC for every module × outcome combination. (B) The full module–phenotype scatter landscape underlying Figure 6c, both predictors across every module and outcome. Leave‐one‐subject‐out sensitivity and the multivariate classifier are reported as sheets in Table S7.

**Figure S8.** Protein‐level detail for the four WGCNA modules carrying an association. One row per module: every assigned protein as a group‐mean z‐score across the four age × timepoint cells (left); the module eigengene for each subject from pre to post in both age groups (center); and the module's most enriched over‐representation terms (right). (A) Turquoise, Lipid Catabolism | TCA Cycle. (B) Black, Glycolysis | Transl. Fact. (C) Yellow, Muscle Contraction | Sarcomere (D) Blue, Protein Folding | Chaperones.

**Figure S9.** Cohort structure and what adjusting for it costs. The four models as successive coarsenings of one partition (A), the proteins each finds per contrast (B), and the standard errors (C), fold‐change agreement (D) and aging *p*‐value distributions (E) behind those counts. Every design reaches full rank; the tables are in Table S10.

## Data Availability

Processed proteomics data are available at DOI 10.5281/zenodo.19886624. Analysis code is available at https://github.com/Dustyn‐T‐Lewis/YvO_2026.
