# Supplementary material for: Aging concomitantly reduces skeletal muscle proteome plasticity and hypertrophic responses to resistance training in male adults
Source: Physiol Rep. 2026 Oct 11;14(19):e71141. doi: 10.14814/phy2.71141 (PMC13655361; doi:10.14814/phy2.71141)
Supplement: Supplementary file 1 [file PHY2-14-e71141-s002.pdf]

**WGCNA modules carrying an age or training association**

**A** **Turquoise: Lipid Catabolism | TCA Cycle (age r = +0.58, q = 2.5e-05)**

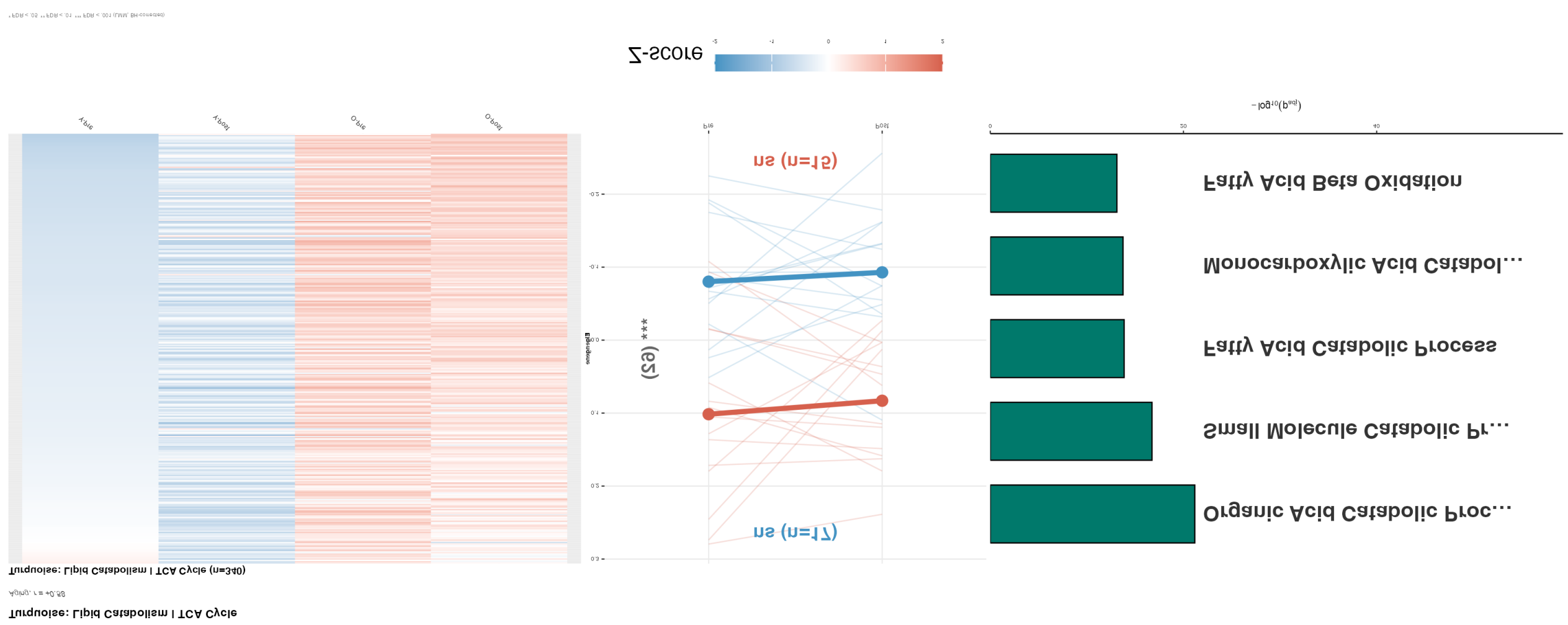


**B** **Black: Glycolysis | Transl. Fact. (age r = -0.60, q = 2.3e-05)**

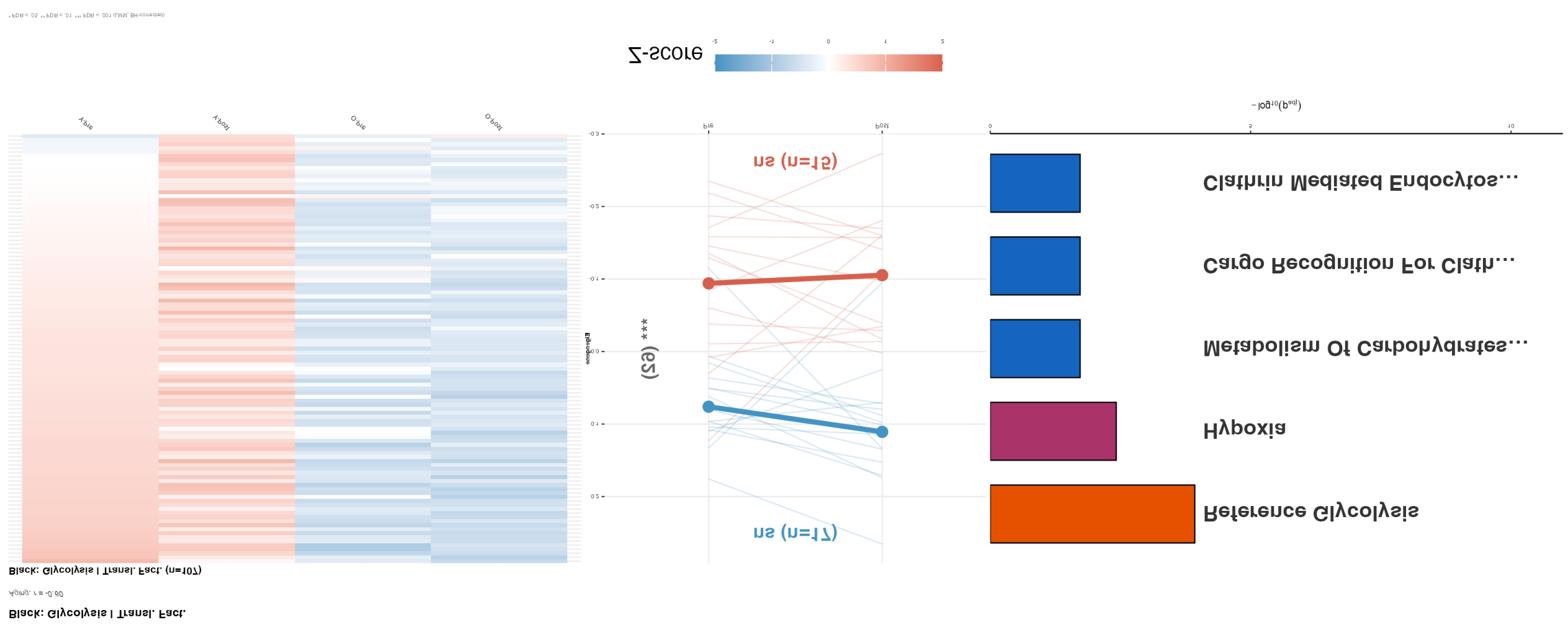


**C** **Yellow: Muscle Contraction | Sarcomere (age r = -0.44, q = 8.1e-03)**

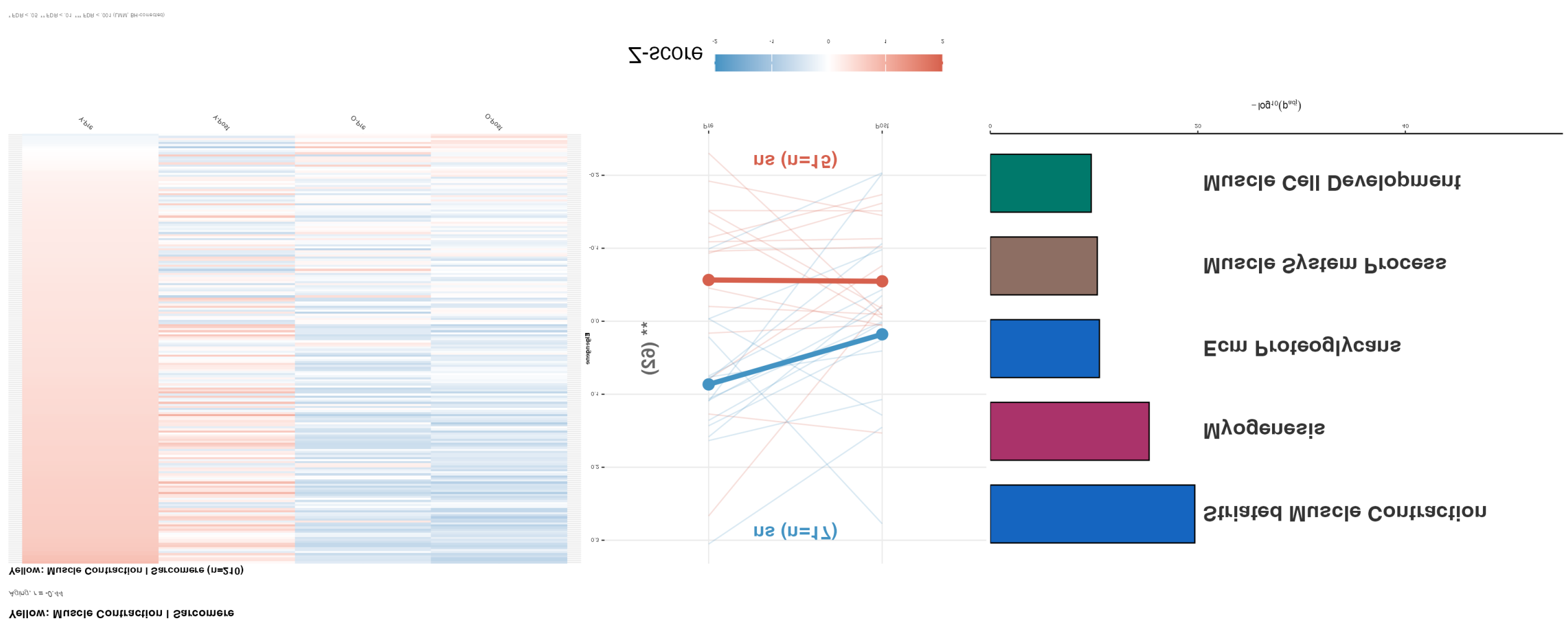


**D** **Blue: Protein Folding | Chaperones (training in the young r = +0.62, q = 2.2e-03)**

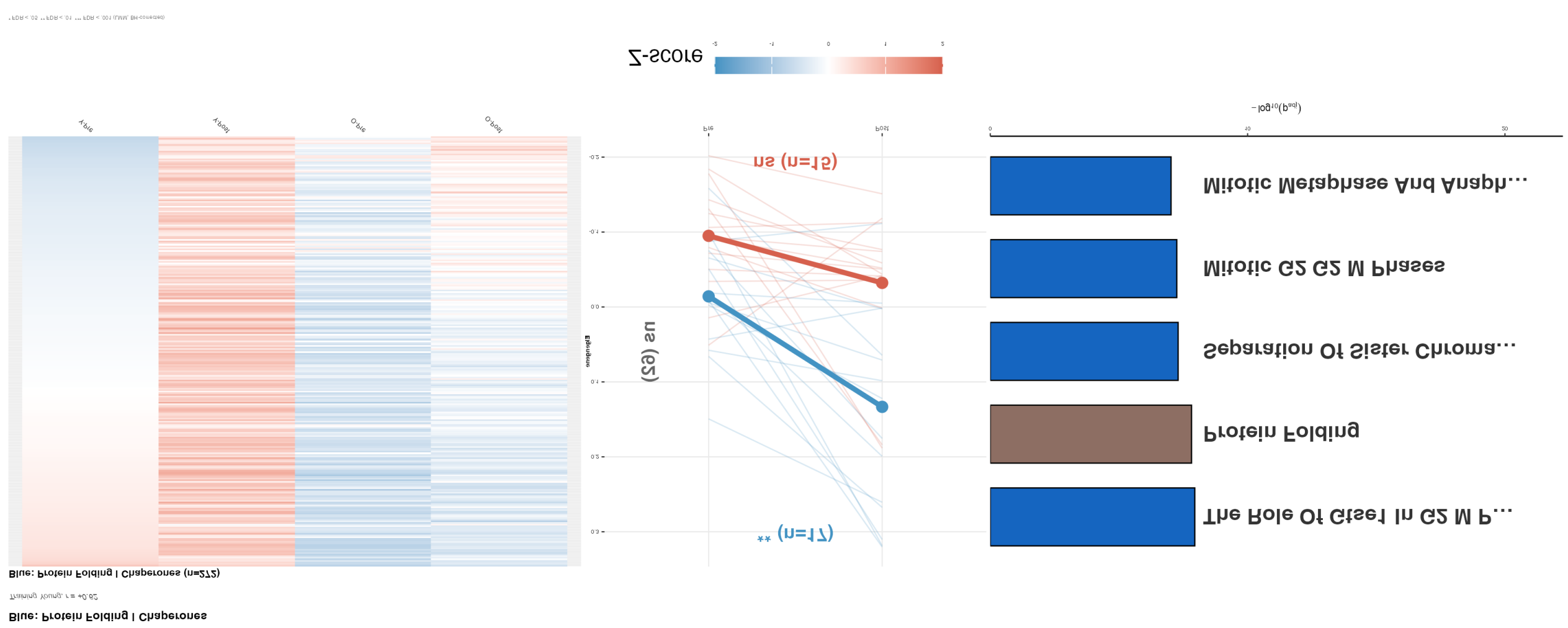


S8 Figure. Protein-level detail for the four WGCNA modules associated with age or training. One row per module: every assigned protein as a group-mean z-score across the four age × timepoint cells (left); the module eigengene for each subject from pre to post in both age groups (center); and the module's most enriched over-representation terms (right). (A) Turquoise, Lipid Catabolism | TCA Cycle. (B) Black, Glycolysis | Transl. Fact. (C) Yellow, Muscle Contraction | Sarcomere (D) Blue, Protein Folding | Chaperones.
