# Supplementary material for: Aging concomitantly reduces skeletal muscle proteome plasticity and hypertrophic responses to resistance training in male adults
Source: Physiol Rep. 2026 Oct 11;14(19):e71141. doi: 10.14814/phy2.71141 (PMC13655361; doi:10.14814/phy2.71141)
Supplement: Supplementary file 2 [file PHY2-14-e71141-s003.pdf]

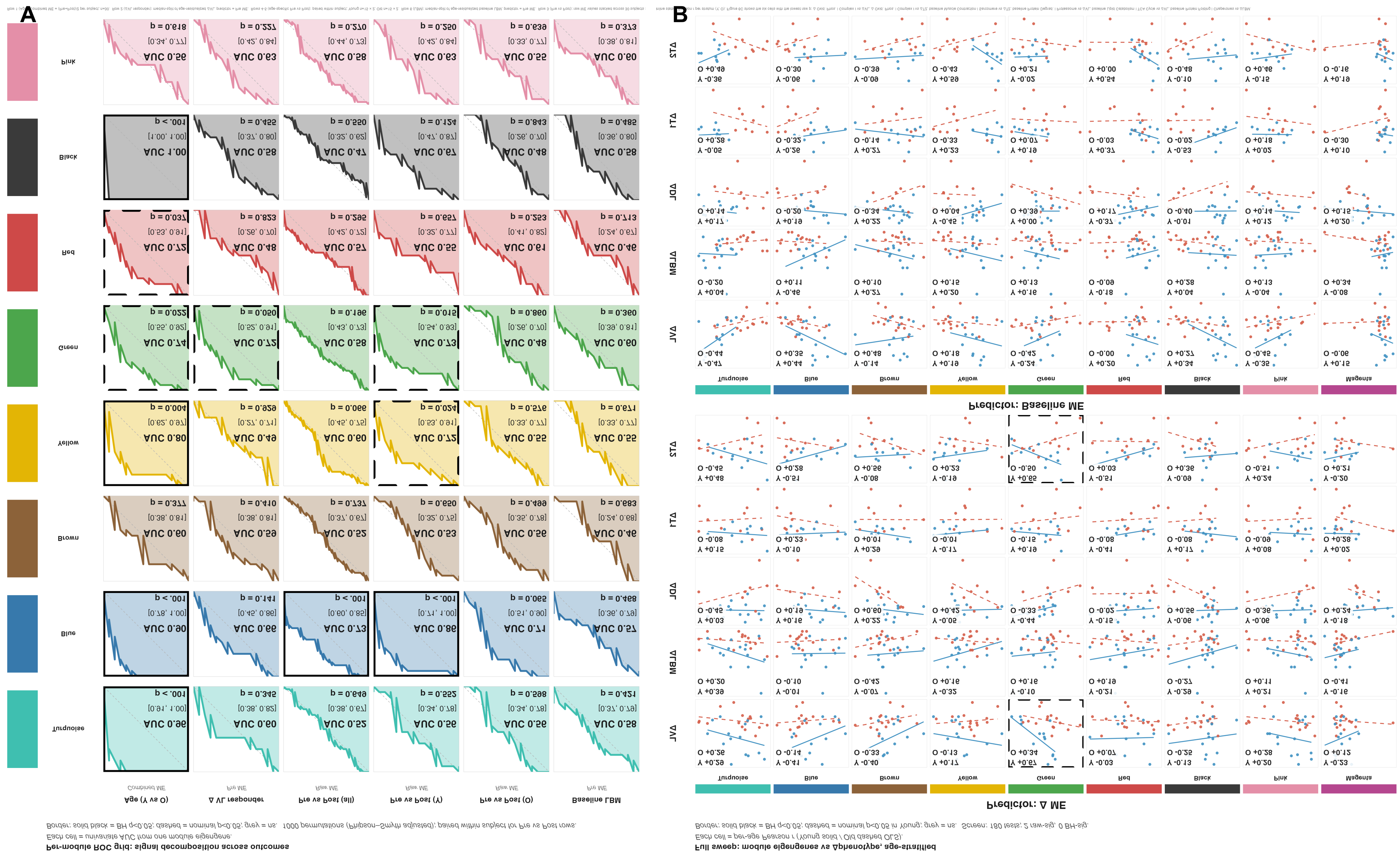

S7 Figure. Module eigengene discrimination and module–phenotype association across the full sweep. (A) Per-module ROC grid: one univariate AUC for every module × outcome combination. (B) Module–phenotype scatter plots for every module and outcome, both predictors, underlying Figure 6C. Leave-one-subject-out sensitivity and the multivariate classifier are reported as sheets in S7 Table.
