# Supplementary material for: Aging concomitantly reduces skeletal muscle proteome plasticity and hypertrophic responses to resistance training in male adults
Source: Physiol Rep. 2026 Oct 11;14(19):e71141. doi: 10.14814/phy2.71141 (PMC13655361; doi:10.14814/phy2.71141)

S4b Figure. Per-protein abundance across the two significance thresholds. Row z-scored abundance per sample, hierarchically clustered within functional groups (GO Slim and Hallmark). (A) Aging at FDR < 0.05. (B) Aging at $\Pi < 0.05$. (C) Training-in-Young at FDR < 0.05. (D) Training-in-Young at $\Pi < 0.05$.

(A) Aging signature: FDR-significant (functional classification)

278 Aging FDR < 0.05 proteins | abundance per sample (row z-score) | hierarchical clustering within 10 functional groups (GO Slim + Hallmark)

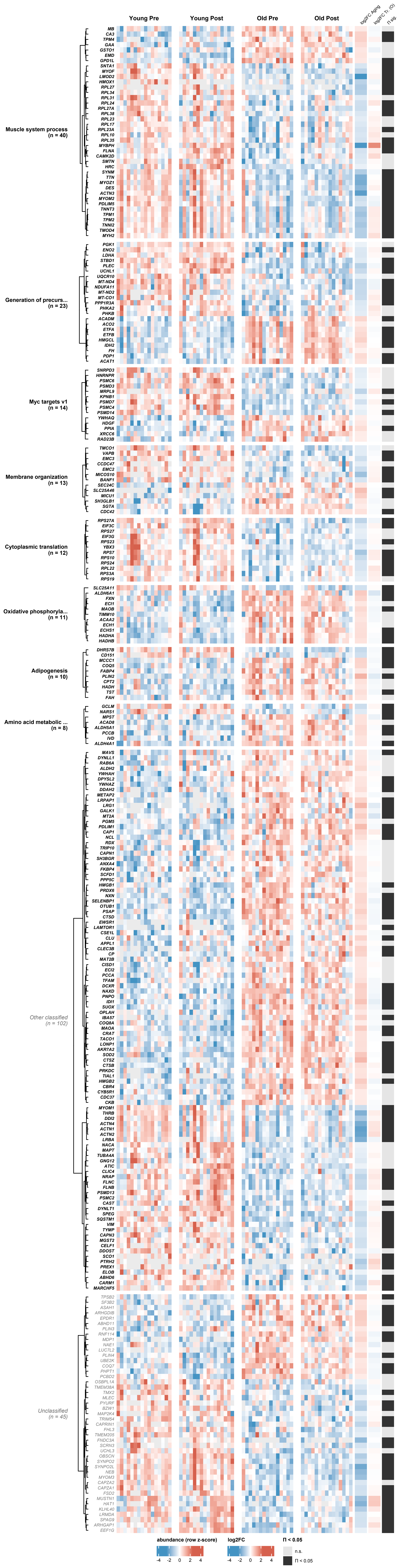

(B) Aging signature: Pi-significant (functional classification)

195 Aging Π < 0.05 proteins | abundance per sample (row z-score) | hierarchical clustering within 9 functional groups (GO Slim + Hallmark)

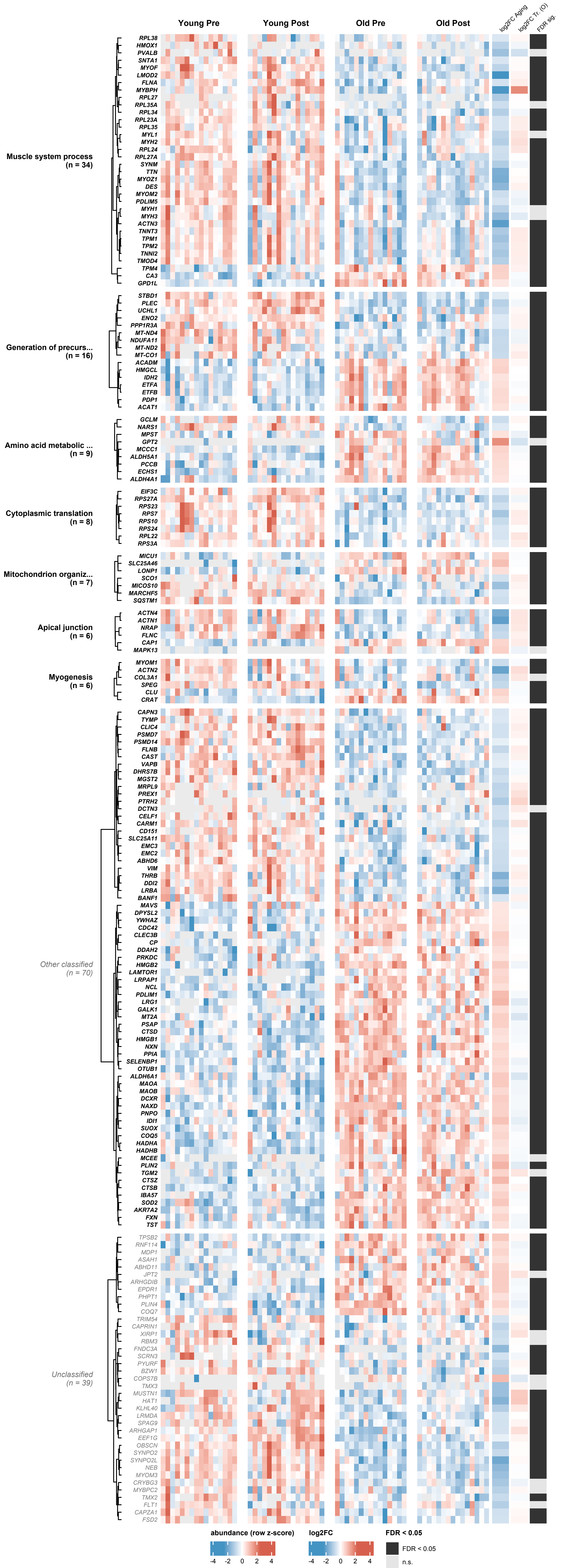

(C) Training (Young) signature: FDR-significant (functional classification)

135 Training(Y) FDR < 0.05 proteins | abundance per sample (row z-score) | hierarchical clustering within 14 functional groups (GO Slim + Hallmark)

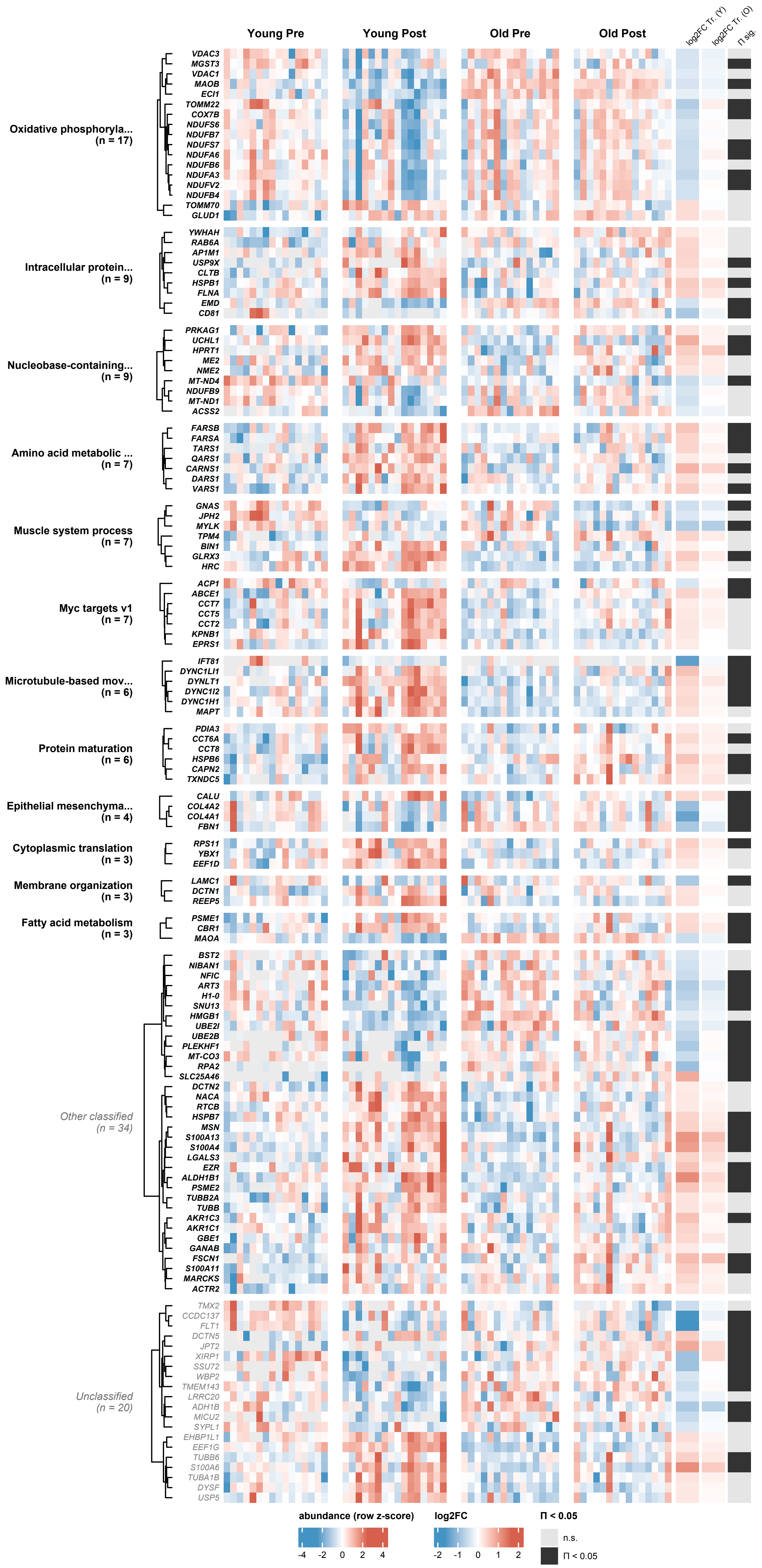

(D) Training (Young) signature: Pi-significant (functional classification)

99 Training(Y) Π < 0.05 proteins | abundance per sample (row z-score) | hierarchical clustering within 15 functional groups (GO Slim + Hallmark)

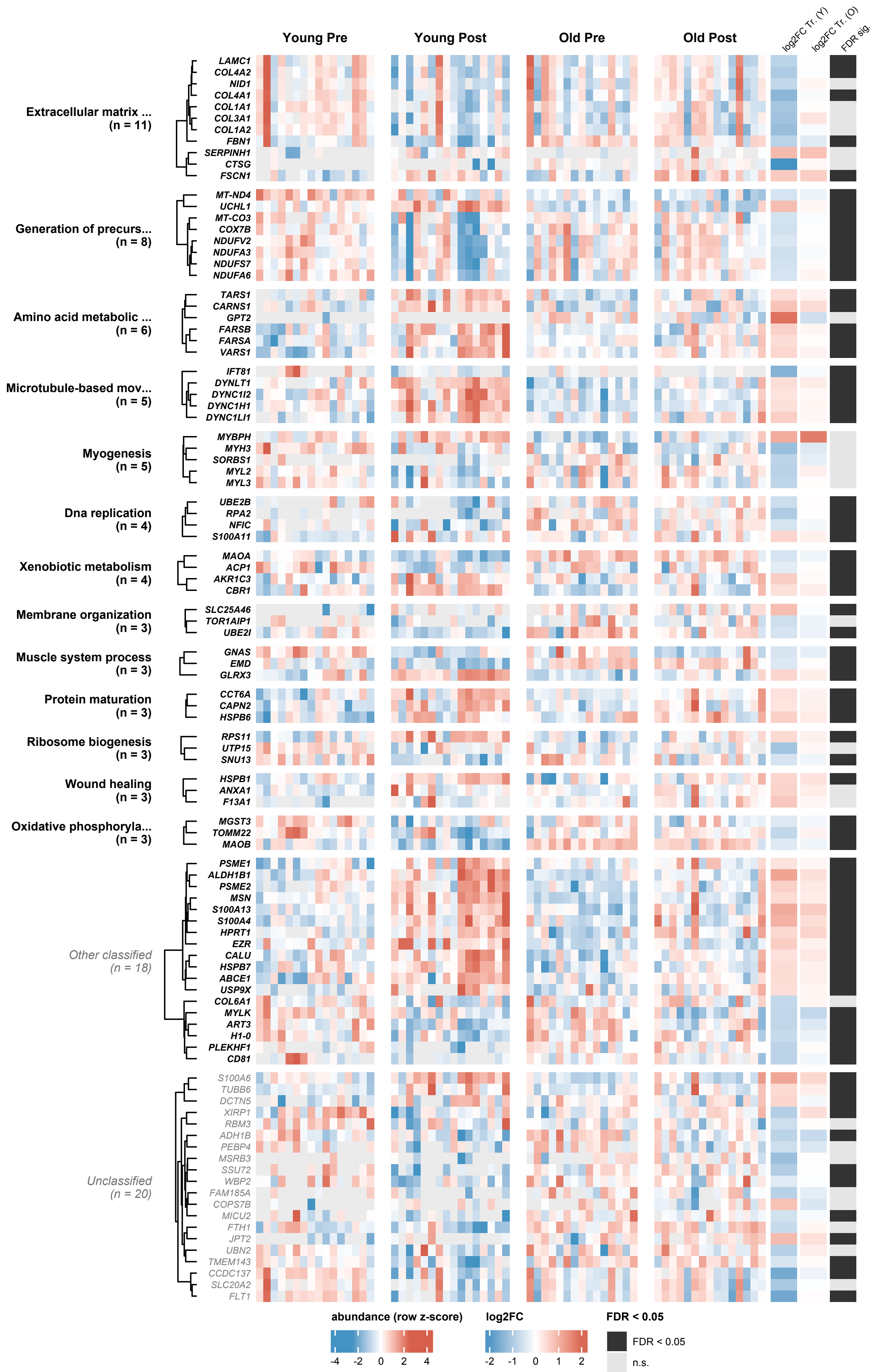

Supplement: Supplementary file 3 [file PHY2-14-e71141-s004.pdf]
