# Supplementary material for: Aging concomitantly reduces skeletal muscle proteome plasticity and hypertrophic responses to resistance training in male adults
Source: Physiol Rep. 2026 Oct 11;14(19):e71141. doi: 10.14814/phy2.71141 (PMC13655361; doi:10.14814/phy2.71141)
Supplement: Supplementary file 4 [file PHY2-14-e71141-s006.pdf]

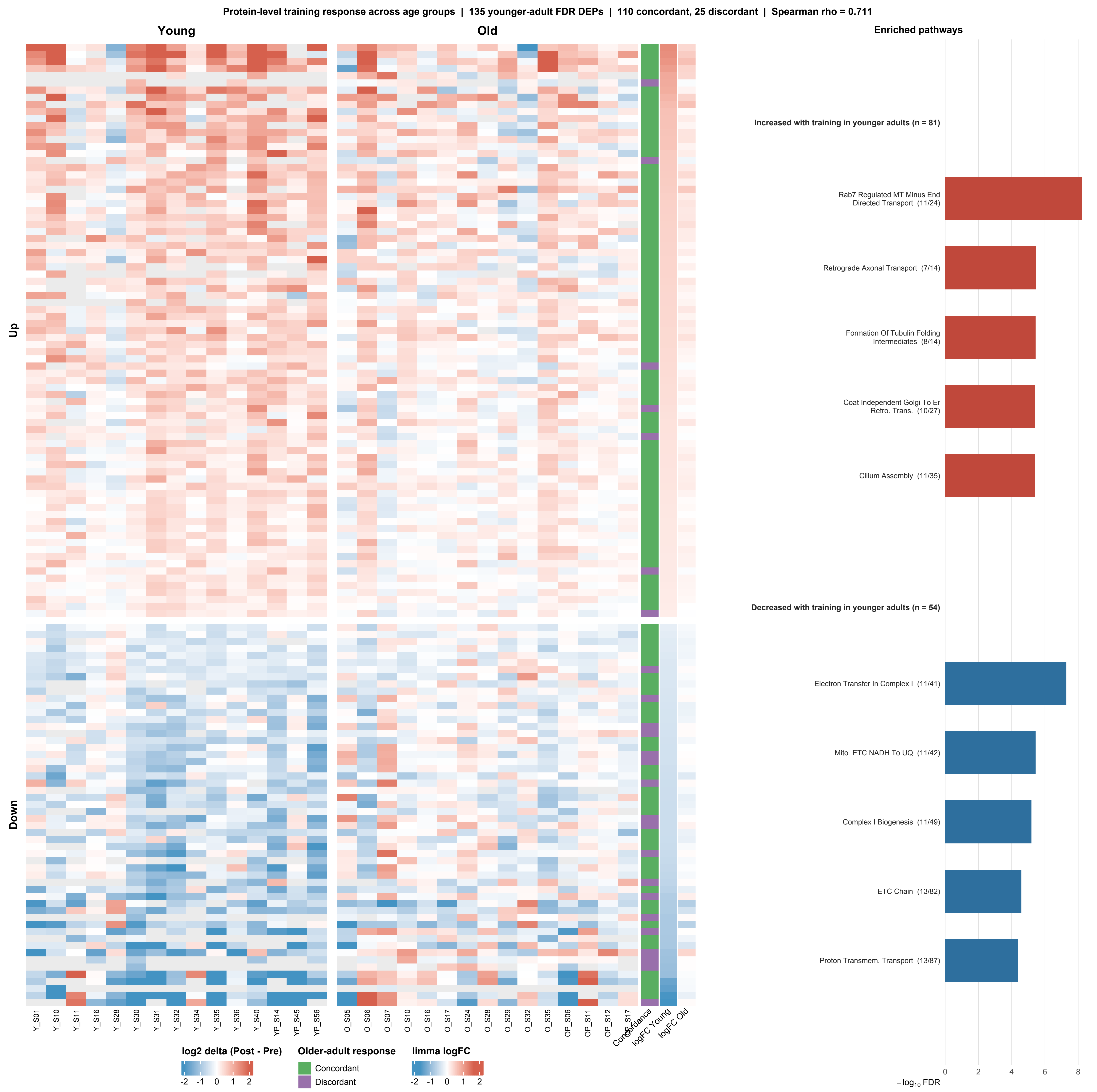

S5b Figure. Protein-level training response across age groups. Per-subject log_2 change (Post – Pre) for the proteins reaching FDR < 0.05 with training in younger adults, across the paired younger and older participants. Rows are grouped by the direction of the younger-adult response and annotated with whether the older-adult response shares that sign; grey cells are proteins not quantified in that participant. The right-hand panel gives the most enriched over-representation terms for the increased and for the decreased sets.
