# Supplementary material for: Aging concomitantly reduces skeletal muscle proteome plasticity and hypertrophic responses to resistance training in male adults
Source: Physiol Rep. 2026 Oct 11;14(19):e71141. doi: 10.14814/phy2.71141 (PMC13655361; doi:10.14814/phy2.71141)
Supplement: Supplementary file 5 [file PHY2-14-e71141-s007.pdf]

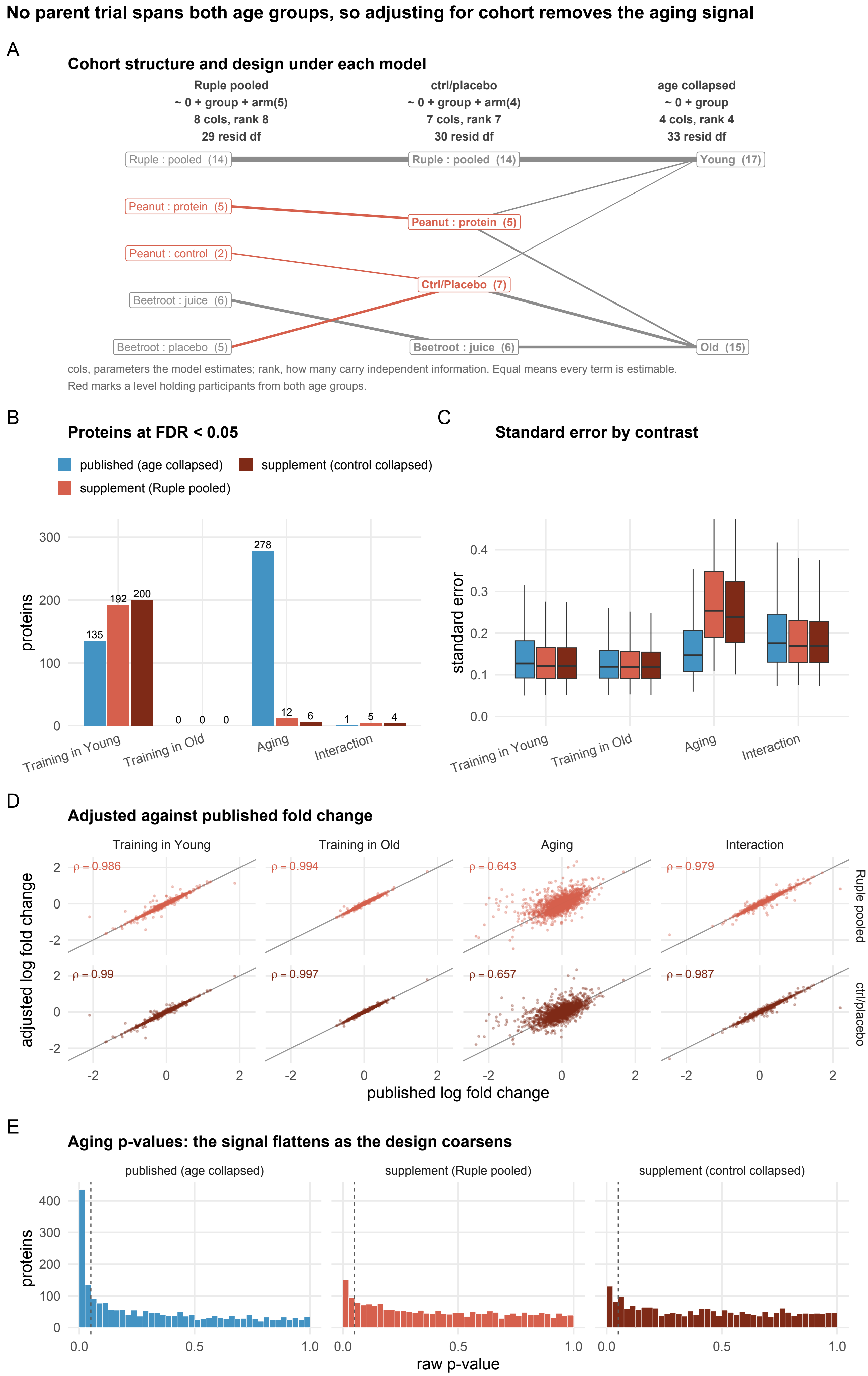

S9 Figure. Cohort structure and the effect of adjusting for it. (A) The four models, from the most detailed cohort grouping to the simplest. (B) Proteins detected per contrast under each model. (C) Standard errors, (D) fold-change agreement and (E) aging p-value distributions behind those counts. All four designs are estimable (full rank); results are in S10 Table.
