# Supplementary material for: Aging concomitantly reduces skeletal muscle proteome plasticity and hypertrophic responses to resistance training in male adults
Source: Physiol Rep. 2026 Oct 11;14(19):e71141. doi: 10.14814/phy2.71141 (PMC13655361; doi:10.14814/phy2.71141)
Supplement: Supplementary file 6 [file PHY2-14-e71141-s008.pdf]

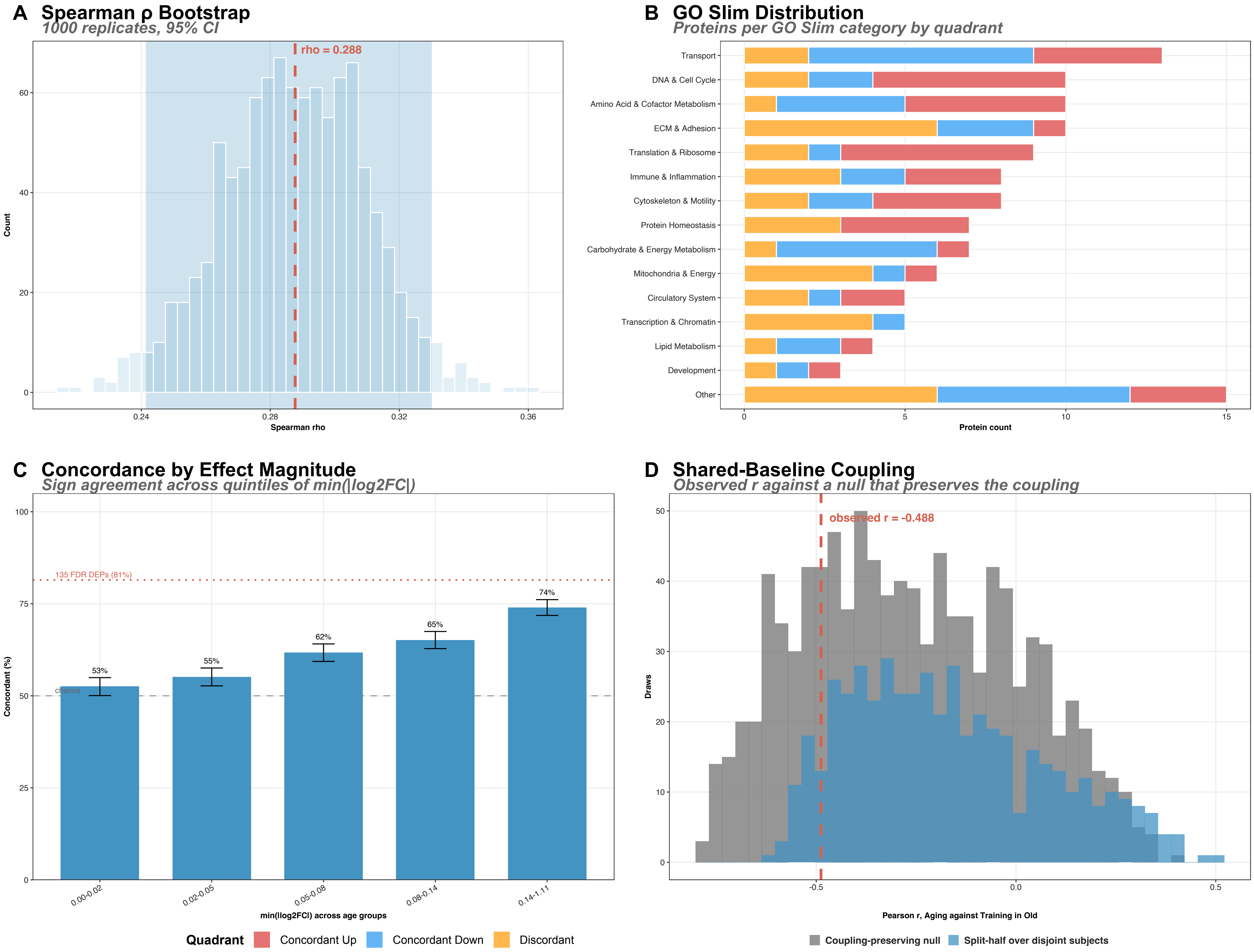

S5a Figure. Training concordance robustness diagnostics. (A) Spearman rho bootstrap. (B) GO Slim category distribution by quadrant. (C) Sign concordance across quintiles of the smaller absolute $\log_2$ fold change. (D) Test of whether the Aging and Training-in-Old correlation arises from their shared baseline samples.
