# Supplementary material for: Aging concomitantly reduces skeletal muscle proteome plasticity and hypertrophic responses to resistance training in male adults
Source: Physiol Rep. 2026 Oct 11;14(19):e71141. doi: 10.14814/phy2.71141 (PMC13655361; doi:10.14814/phy2.71141)
Supplement: Supplementary file 7 [file PHY2-14-e71141-s011.pdf]

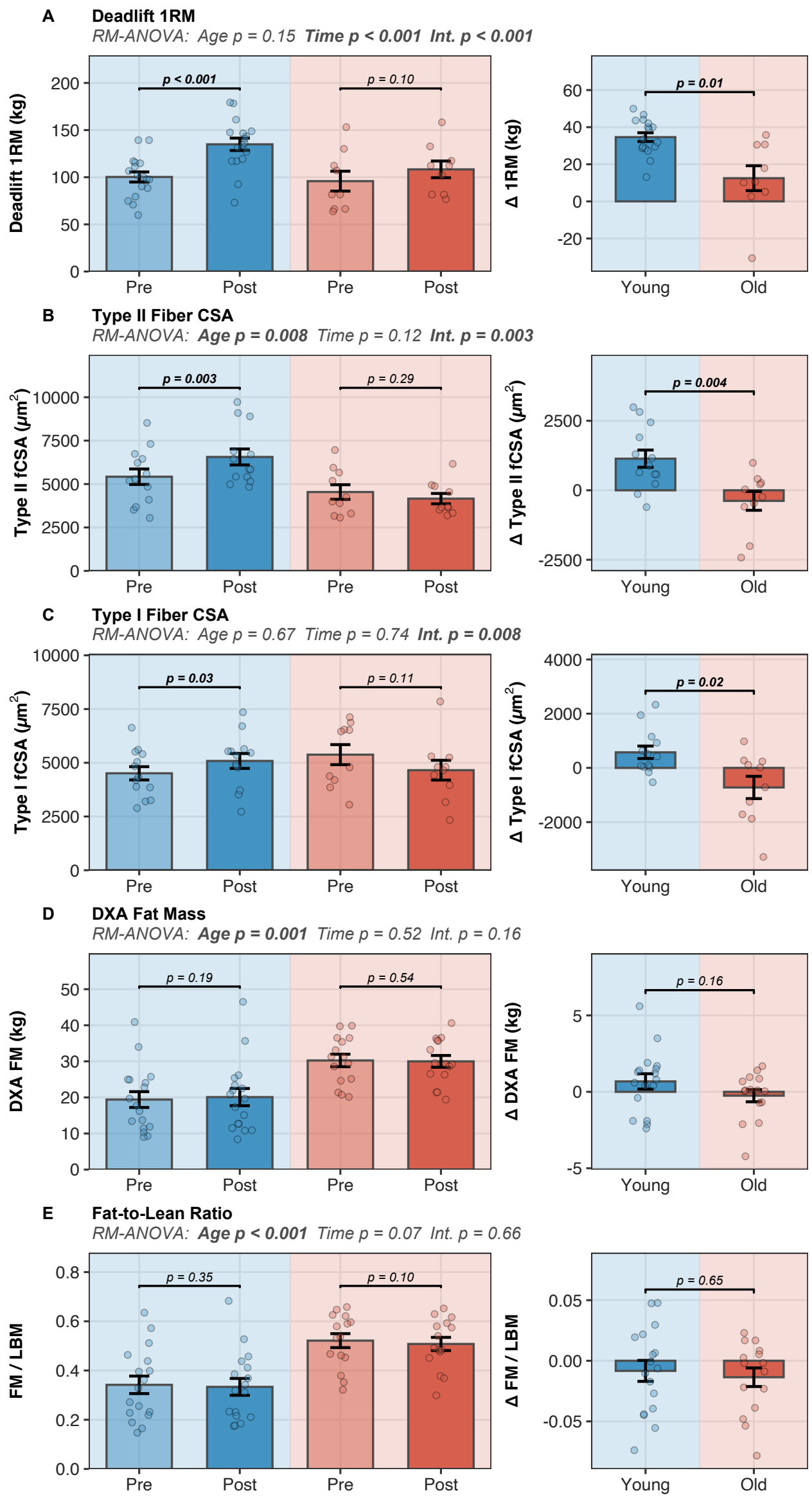

S2 Figure. Phenotypic responses to resistance training by age group. (A) Deadlift one-repetition maximum. (B) Type II fiber cross-sectional area. (C) Type I fiber cross-sectional area. (D) Whole-body DXA fat mass. (E) Fat-to-lean mass ratio.
