# Supplementary material for: Aging concomitantly reduces skeletal muscle proteome plasticity and hypertrophic responses to resistance training in male adults
Source: Physiol Rep. 2026 Oct 11;14(19):e71141. doi: 10.14814/phy2.71141 (PMC13655361; doi:10.14814/phy2.71141)
Supplement: Supplementary file 8 [file PHY2-14-e71141-s012.pdf]

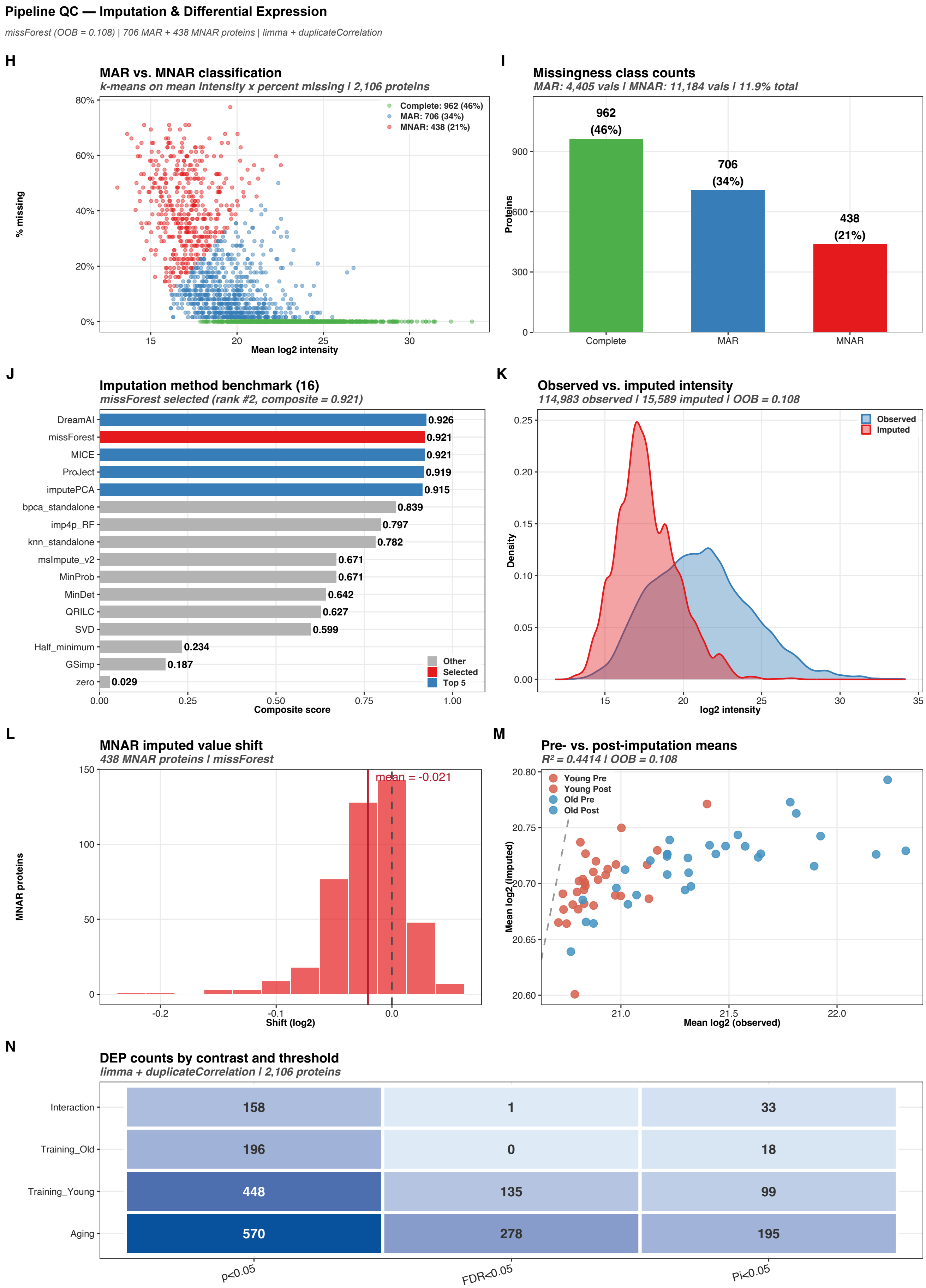


S1b Figure. Imputation diagnostics. (H) Missingness classification scatter (MAR vs MNAR). (I) Missingness classification counts. (J) 16-method imputation benchmark (OOB NRMSE = 0.108). (K) Imputation density overlay. (L) MNAR shift diagnostic. (M) Sample integrity post-imputation. (N) DEP sensitivity heatmap.
