# Supplementary material for: Aging concomitantly reduces skeletal muscle proteome plasticity and hypertrophic responses to resistance training in male adults
Source: Physiol Rep. 2026 Oct 11;14(19):e71141. doi: 10.14814/phy2.71141 (PMC13655361; doi:10.14814/phy2.71141)
Supplement: Supplementary file 9 [file PHY2-14-e71141-s015.pdf]

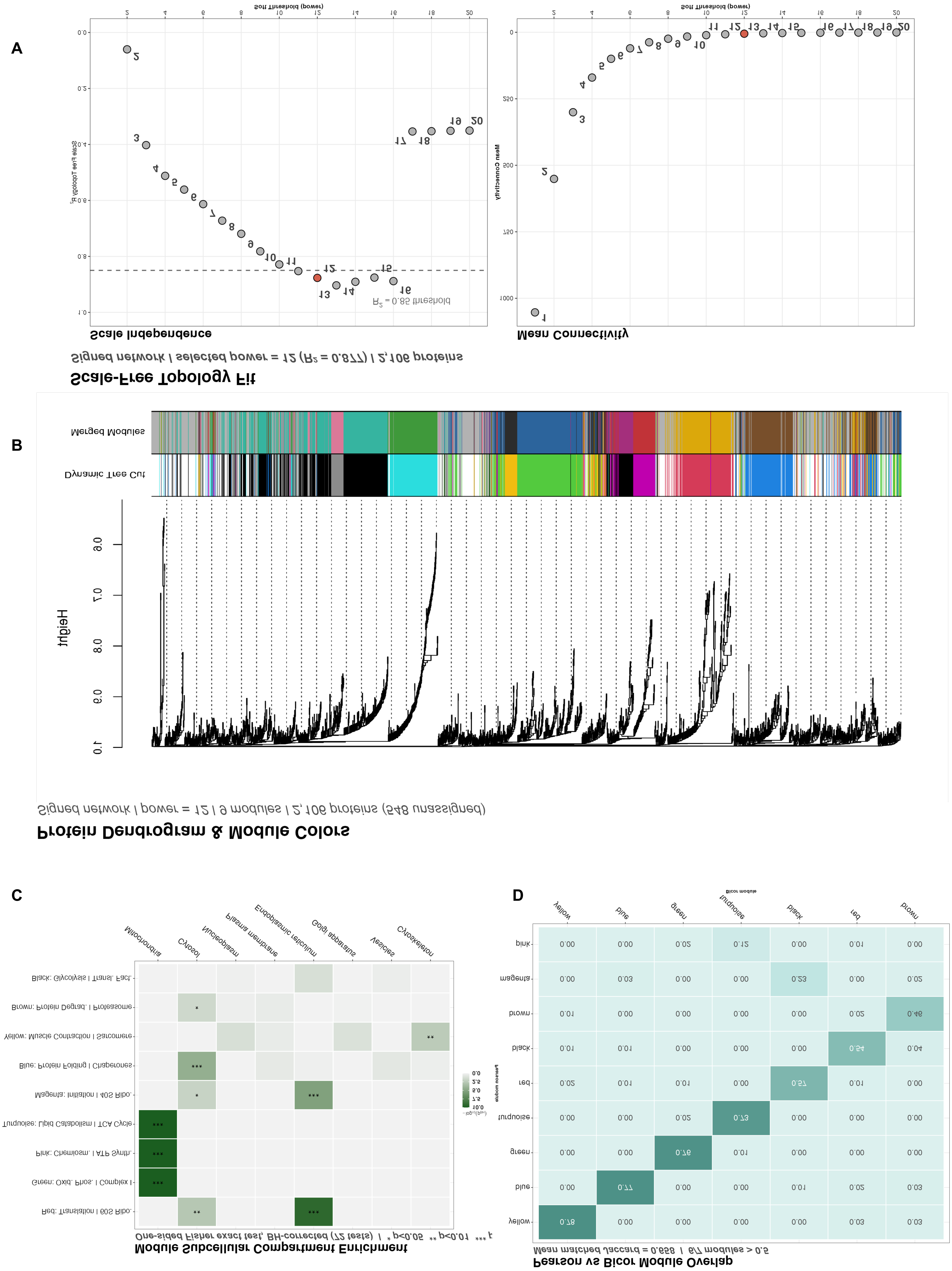

S6 Figure. WGCNA network construction diagnostics. (A) Scale-free topology fit. (B) Protein dendrogram with module assignments. (C) Subcellular compartment enrichment. (D) Pearson vs. biweight midcorrelation module overlap.
