# Supplementary material for: Aging concomitantly reduces skeletal muscle proteome plasticity and hypertrophic responses to resistance training in male adults
Source: Physiol Rep. 2026 Oct 11;14(19):e71141. doi: 10.14814/phy2.71141 (PMC13655361; doi:10.14814/phy2.71141)
Supplement: Supplementary file 10 [file PHY2-14-e71141-s016.pdf]

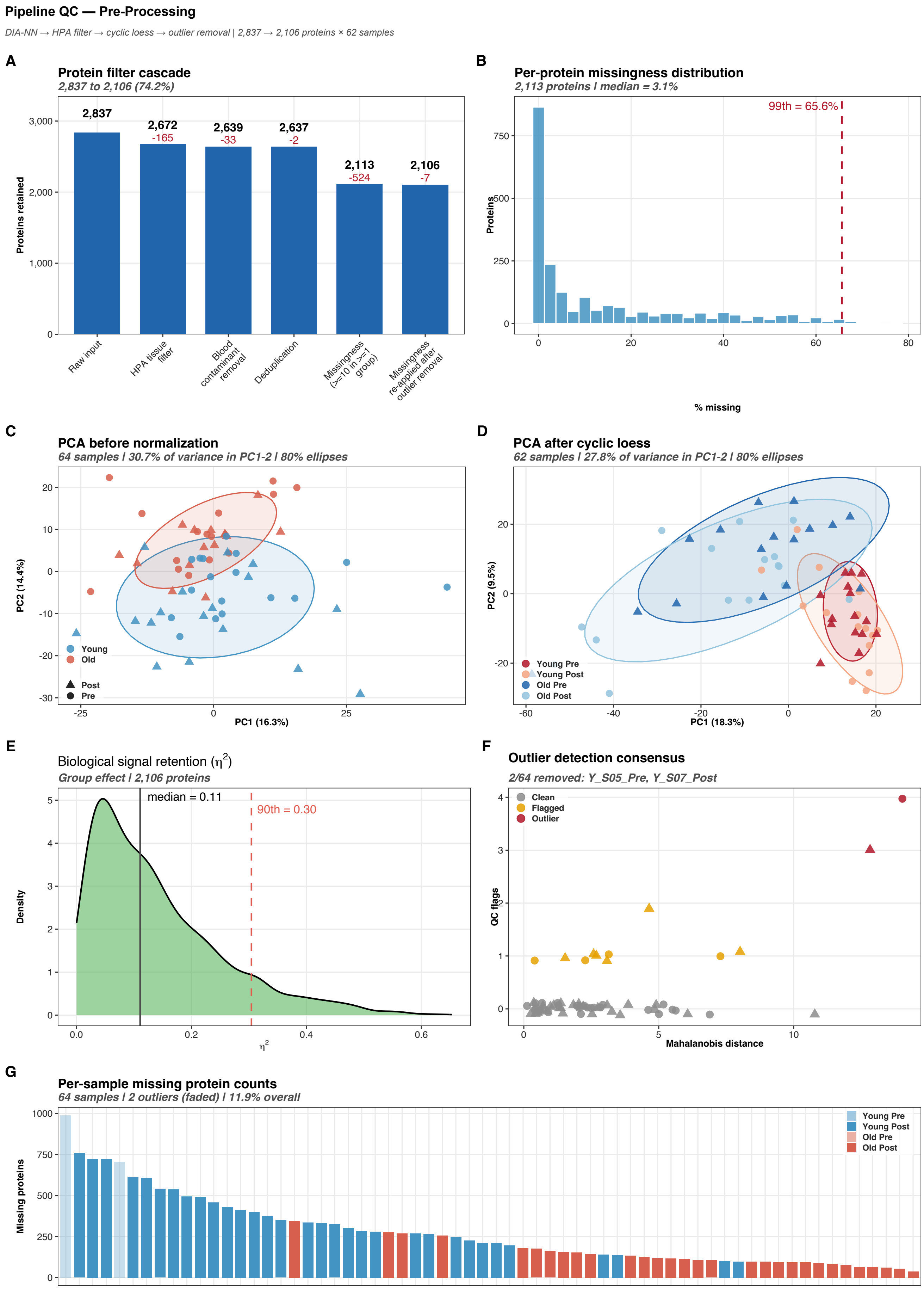

S1a Figure. Quality-control and normalization pipeline. (A) Protein filter cascade (2,837 to 2,106). (B) Per-protein missingness. (C) Pre-normalization PCA. (D) Post-normalization PCA. (E) Eta-squared variance decomposition. (F) Outlier consensus scoring. (G) Per-sample missingness.
