# Supplementary material for: Aging concomitantly reduces skeletal muscle proteome plasticity and hypertrophic responses to resistance training in male adults
Source: Physiol Rep. 2026 Oct 11;14(19):e71141. doi: 10.14814/phy2.71141 (PMC13655361; doi:10.14814/phy2.71141)
Supplement: Supplementary file 11 [file PHY2-14-e71141-s017.pdf]

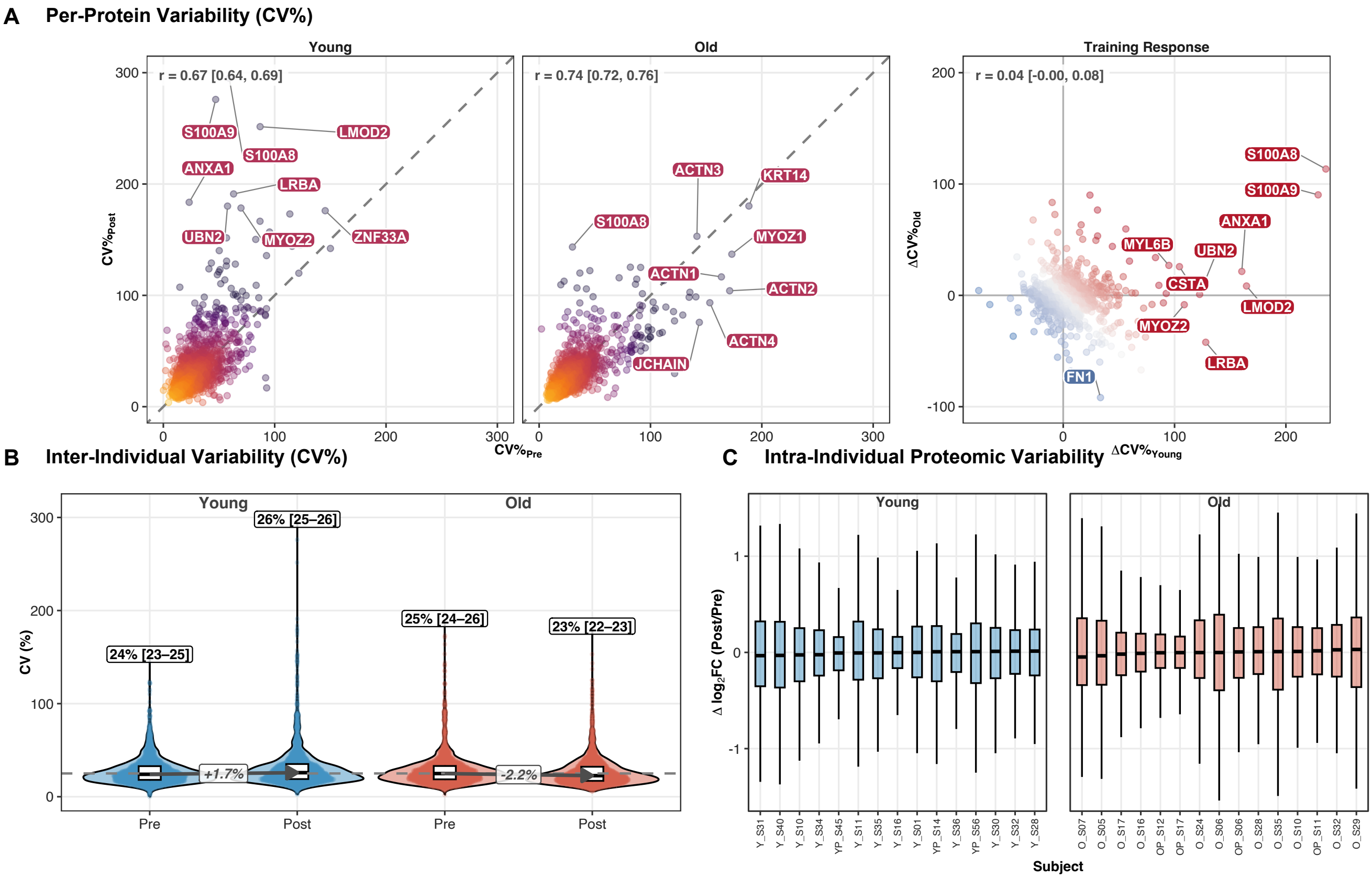

S3 Figure. Proteome-level coefficient of variation by experimental group. (A) Per-protein variability (CV%). (B) Inter-individual variability (CV%). (C) Intra-individual proteomic variability across the imputed matrix.
