# Supplementary material for: Aging concomitantly reduces skeletal muscle proteome plasticity and hypertrophic responses to resistance training in male adults
Source: Physiol Rep. 2026 Oct 11;14(19):e71141. doi: 10.14814/phy2.71141 (PMC13655361; doi:10.14814/phy2.71141)
Supplement: Supplementary file 12 [file PHY2-14-e71141-s021.pdf]

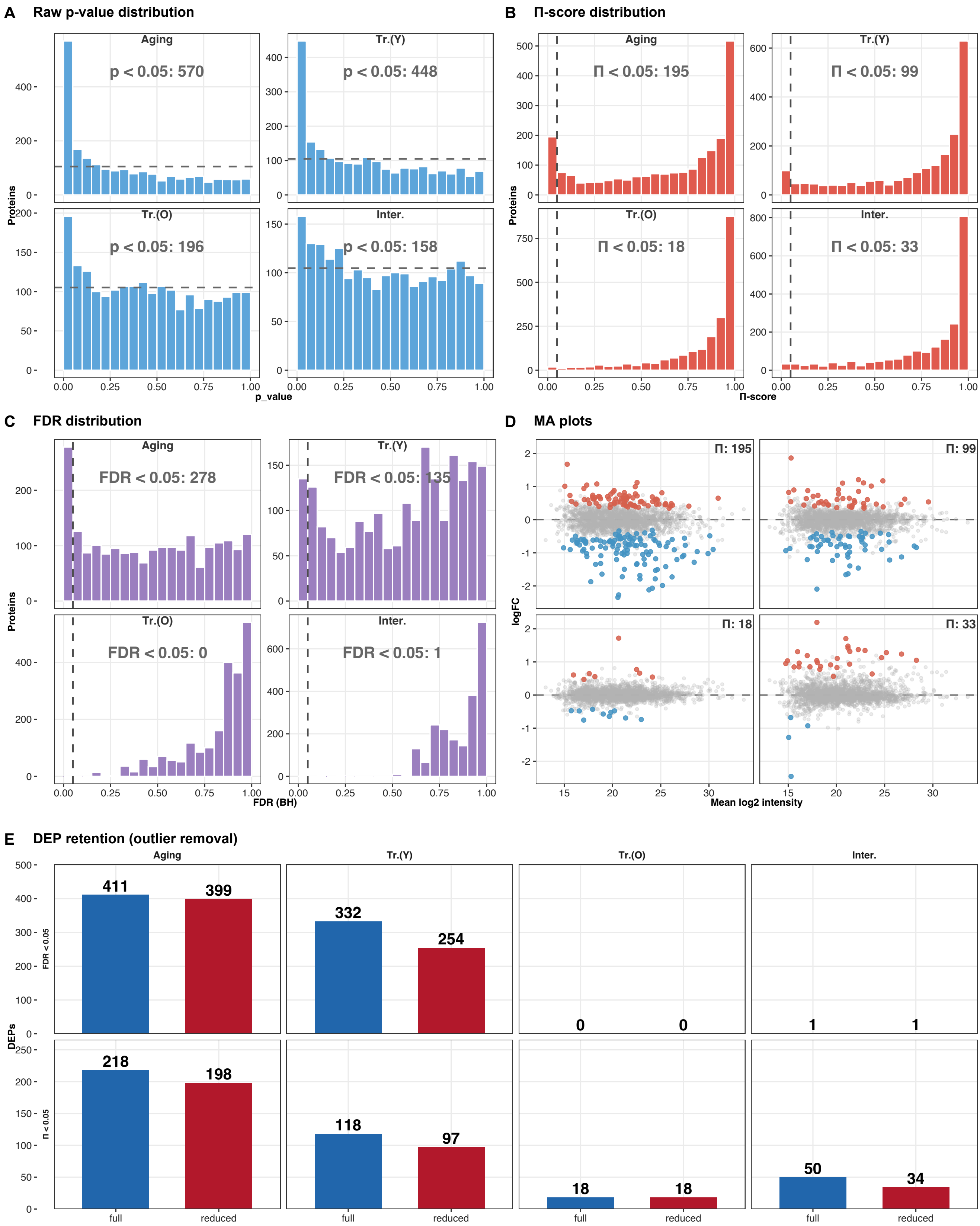

S4a Figure. Differential expression model diagnostics across contrasts. (A) P-value histograms. (B) Pi-score distributions. (C) FDR distributions. (D) MA plots. (E) DEP retention after outlier removal.
